# Protocol of a multi-centre randomized controlled trial to compare pericapsular nerve group block, fascia-iliaca compartment block and femoral nerve block for pain management in patients with a hip fracture in the emergency department (CPFF-ED)

**DOI:** 10.1371/journal.pone.0342422

**Published:** 2026-02-09

**Authors:** Jurian Dolstra, Svenja L. Haak, E. Christiaan Boerma, Heleen Lameijer, Ewoud Ter Avest

**Affiliations:** 1 Department of Emergency Medicine, Frisius Medical Centre Leeuwarden, Leeuwarden, The Netherlands; 2 Department of Sustainable Health, University of Groningen, Campus Fryslân Leeuwarden, Leeuwarden, The Netherlands; 3 Department of Acute Care, University of Groningen, University Medical Centre Groningen, Groningen, The Netherlands; 4 Department of Intensive Care, Frisius Medical Centre Leeuwarden, Leeuwarden, The Netherlands; 5 London’s Air Ambulance, London, United Kingdom; Menoufia University, EGYPT

## Abstract

**Background:**

Patients presenting in the emergency department (ED) with a hip fracture are often in significant pain. Opioids are frequently prescribed as part of standardized pain protocols, but are associated with many adverse events, especially in the elderly. Regional anaesthesia with a femoral nerve block (FNB), Fascia Iliaca compartment block (FICB) or Pericapsular nerve block (PENG) may provide a reasonable alternative to these patients. We designed a prospective multicentre study to compare the efficacy and safety of these blocks in the ED setting, which has been registered in the Centrale Commissie Mensgebonden Onderzoek (CCMO) Research Portal (NL-OMON57433).

**Methods and analysis:**

A multicentre Randomized Controlled Trial (RCT), conducted in the ED of 5 hospitals in the Netherlands. A total of 254 adult patients (18+) presenting to the ED with a neck of femur or pertrochanteric femur fracture will be randomised to receive FNB, FICB or PENG in the ED after informed consent has been obtained. Patients will be followed for 12 hours after block placement in the ED. The primary (patient reported) outcome measure is overall patient wellbeing 6–8 hours after block administration (or until operation, whichever comes first) as scored by the validated Quality of Recovery (QoR-15) questionnaire. Secondary outcome measures will be pre-operative pain scores, the cumulative dose of analgesics used during the first 12 hours after block placement or until operation, and complication rates for each of the blocks.

**Expected results:**

This randomized controlled trial with a superiority design will allow conclusions on which regional nerve block is most effective in improving pre-operative wellbeing in patients with a neck of femur or pertrochanteric fracture. The choice to use a patient reported outcome measure to evaluate block efficacy maximizes clinical relevance. And by allowing the choice of local anaesthetic to the participating hospital (with a 1:1:1 randomization for each participating centre) clinical applicability of our findings is maximized.

## Introduction

With an aging population, the number of patients presenting to the emergency department (ED) with a hip fracture increases each year [1,2]. Most hip fractures require surgical treatment. To improve patient wellbeing in the period from presentation until surgery adequate pain management is of utmost importance, as pain is not only unpleasant but also an important factor affecting outcomes [3–5]. Therefore, systemic analgesia with NSAIDS or opioids is commonly provided [6]. However, especially opioids are associated with unwanted adverse events such as delirium, vomiting, pruritus and respiratory depression [7,8]. Additionally, opioids are highly addictive [9].

Regional anaesthesia may provide a reasonable alternative to provide analgesia to patients with a hip fracture. It has the potential to provide analgesia without the unwanted opioid-related adverse events [5,10–13]. Previous research has shown that regional anaesthesia is positively associated with survival and a shorter length of stay in hospital. Various international guidelines therefore endorse their use [14,15].

For regional anaesthesia after hip fractures, different block techniques can be used: Femoral nerve block (FNB), fascia Iliaca compartment block (FICB) or pericapsular nerve block (PENG) So far, literature directly comparing the efficacy and safety of the different nerve block techniques is scarce [16]. Previous studies have not focussed on early administration of the blocks in the ED and/or have looked at pain reduction only, and not on more comprehensive patient-centred outcome measures [16].

The aim of this study is therefore to compare the efficacy and safety of preoperatively placed PENG, FICB and FNB by assessing patient reported outcomes, pain and opioid use in patients with hip fractures presenting in the ED.

## Materials and methods

### Study design and setting

**Fig 1:**
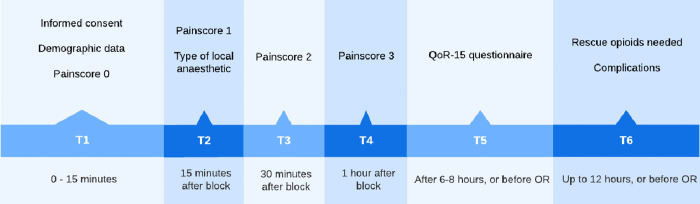
SPIRIT schedule of enrolment, intervention and assessment.

**Fig 2:**
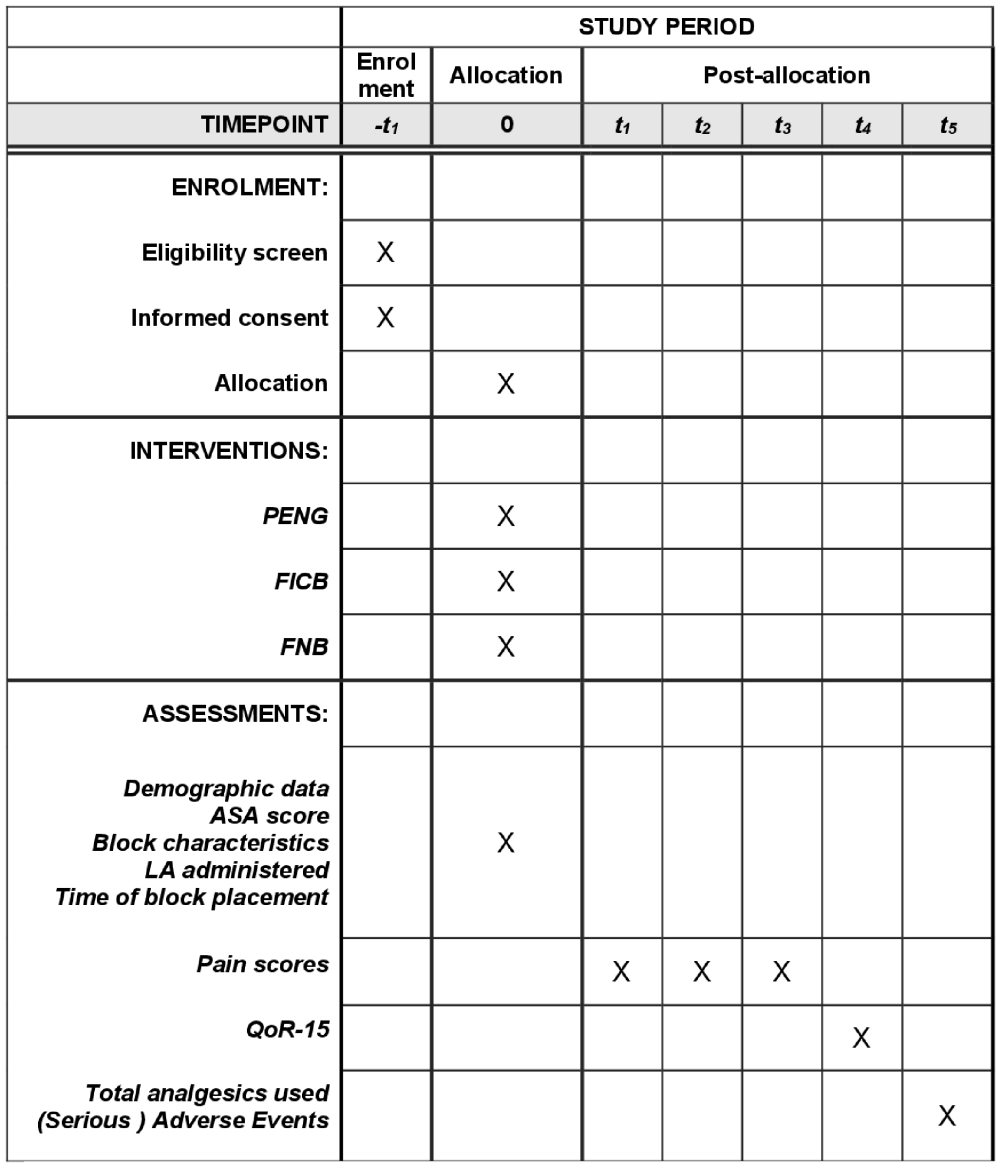
Flowchart of the study design.

We aim to conduct a multicentre Randomized Controlled Trial (RCT), in the ED of 5 hospitals in the Netherlands. Patients with a neck of femur or pertrochanteric fracture are randomized to receive one of the following nerve blocks: FICB, FNB or PENG. After randomization and obtaining informed consent, regional anaesthesia is provided according to a standardized protocol (see below) and pain scores, analgesic use and adverse events are registered at fixed time intervals. General patient wellbeing, as the main patient centred outcome of the study, will be measured with a validated Quality of Recovery (QoR-15) questionnaire 6–8 hours after block placement or before admittance to the operating room (OR) (whichever one comes first) (Figs 1 and 2).

### Study population

#### Inclusion criteria.

The study population will consist of adult patients (**≥**18 years) with a neck of femur or pertrochanteric femur fracture. To be eligible for inclusion, a (X-ray or CT-confirmed) fracture must be present and the patient should be able and willing to provide informed consent and reliably report symptoms to the research team.

#### Exclusion criteria.

Patients who are allergic to local anaesthetics, have an infection at the injection-site, have a hip fracture in combination with other fractures or visceral injuries warranting (operative) treatment (poly-trauma patients), or who have a subtrochanteric or periprosthetic fracture or a skin injury, will be excluded from participation in this study.

### Sample size calculation

The study sample size is based on the primary outcome measure (QoR-15 score) and a superiority design. Based on previous research from the creators of the QoR-15 questionnaire, a 6-point difference in QoR-15 score is deemed of clinical relevance [17]. Assuming a QoR-15 standard deviation of 10, based on previous literature in which QoR-15 scores are measured in patients receiving either PENG, FICB or FNB [18,19], 231 patients are needed (distributed over three groups of 77) in order to detect a 6-point difference between any of the three groups with a power of 90% and a Bonferroni corrected alpha of 0.0167 to account for multiple testing. To account for 10% attrition, i.e., potential dropouts during the study process, a total of 254 patients will be included.

### Randomization

Patients will be randomized to receive one of three regional nerve block types: FNB, FICB, or PENG. Block-randomization will be performed on a 1:1:1 basis per participating centre using opaque sealed envelopes, with each centre being a stratum using variable block sizes, to account for variation in the type of local anaesthetics being used in each participating centre (see below). No stratification per fracture type is performed.

### Nerve block procedures

Nerve blocks will be performed with either ropivacaine or levobupivacaine depending on the centre where the patient will be presented. Local anaesthetic (LA) dosing will be weight-based and participating hospitals are obliged to adhere to the dosing recommendations in Table 1. In line with existing guidelines, high-volume low-concentration blocks are prescribed per protocol for FICB and PENG blocks, with a minimum volume of 40 ml.

**Table 1:** Dose recommendations of local anaesthetics.

	Levobupivacaine	Ropivacaine
Indicative dosage		
Weight of 50–60 kg	75mg	112,5 mg
Weight of >60 kg*	100mg	150mg

*Until a maximum weight of 100 KG

All blocks will be performed with the patient in a supine position using ultrasound guidance adhering to the procedures as described by the New York Society of Regional Anesthesia (NYSORA) procedures [20–22].

For FNB, the ultrasound transducer is placed transversely on the inguinal crease and moved in a lateral-to-medial direction to identify the femoral artery. The needle tip is placed immediately adjacent to the lateral aspect of the femoral nerve, below the fascia iliaca, that surrounds the femoral nerve and (after negative aspiration) approximately the LA), is injected after negative aspiration. Proper deposition of local anaesthetic is confirmed either by observation of the femoral nerve being displaced by the injectate or by the spread of the local anaesthetic above or below the nerve, surrounding and separating it from the fascia iliaca layers.

For FICB, the femoral artery is visualized by placement of the transducer transversely on the inguinal crease. The needle tip is placed under the fascia iliaca approximately at a lateral third of the line connecting the anterior superior iliac spine to the pubic tubercle. After negative aspiration the local anaesthetic is injected, resulting in the separation of the fascia iliaca by the local anaesthetic in the medial–lateral direction from the point of injection as described.

For PENG, the ultrasound probe is initially placed in a transverse plane over the anterior inferior iliac spine before being aligned with the pubic ramus by rotating the probe 45 degrees counter clockwise. The iliopubic eminence, the iliopsoas muscle tendon, the femoral artery and pectineus muscle should be visualised in this view. The needle tip is placed in the musculofascial plane between the psoas tendon anteriorly and the pubic ramus posteriorly, and the local anaesthetic is injected after negative aspiration.

Participating centres are free to use their own pain relief protocols to co-administer systemic analgesics during the study when the block does not provide sufficient pain relief. Dosages of systemic (rescue) analgesics administered will be recorded for the study.

### Study procedures

#### Training.

Competence of participating physicians in performing all three different nerve blocks is ensured before the start of the study. When physicians perform all three blocks regularly and without supervision, they are eligible to participate. For physicians who have not yet obtained the desired competence level in one or two of the blocks but who are proficient in at least one ultrasound-guided block, competence is attained by offering block procedures under supervision of an expert. Expert-supervised blocks will be performed and recorded in a training log, and only after a minimum of 5 expert-supervised blocks have been performed, eligibility to perform blocks for the study is obtained.

#### Screening and enrolment.

Patients will be screened for eligibility upon their arrival in the ED. If a patient meets eligibility criteria, written informed consent for participation will be obtained.

### Data collection procedure

**Fig 3:**
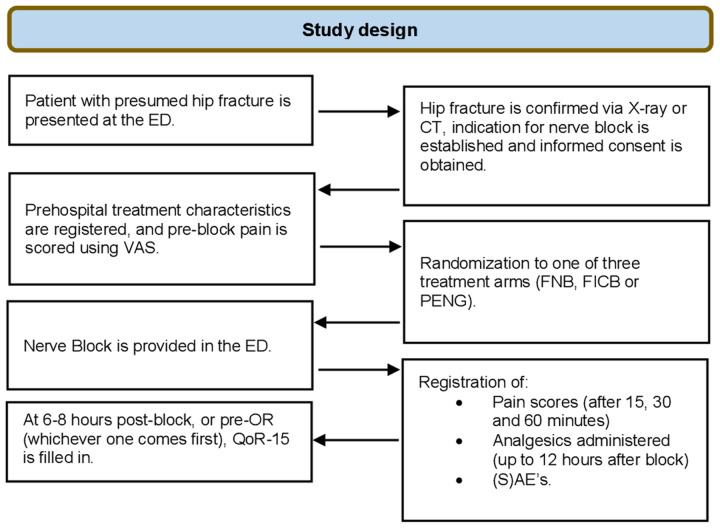
Timeline of the study procedure, showing which data will be collected at what time point.

Data will be gathered at 7 different points in time (T1-T6) (Fig 3).

- At inclusion, the following baseline characteristics will be registered in an electronic case report form (eCRF): (demographic data (age, sex, weight)), ASA score, pain scores (Visual Analog Scale; VAS) in rest and during movement of the affected leg, fracture type, analgesics administered or taken before hospital admission (including medication routinely used by the patient), block characteristics (type of block the patient was randomized to, amount- and type of LA administered, and the exact time of block placement).
- Fifteen, 30 and 60 minutes after block placement, adequacy of block placement will be evaluated by registering pain scores. These pain scores will be registered by ED nurses not involved in the study team to minimize the potential for measurement bias.
- Six to eight hours after block placement the QoR-15 score (primary endpoint) will be collected. If surgery is scheduled to be within this window, QoR-15 will be scored pre-OR. To ensure that patients will be adequately rested for their operation, patients will not be approached to fill in the QoR-15 between 23:00 and 6:00 hours. Therefore, for blocks placed between 17.00 and 22.00, this will be postponed until the next morning.
- Up to 12 hours after block placement (or pre-OR whichever comes first), the total amount of analgesics used and any ((serious) adverse events that occurred after block placement will be registered.

### Ethics approval and informed consent

#### Ethics approval.

This study was reviewed and approved by the Medical ethical review board of the Frisius MC (Regionale Toetsingscommissie Patiëntgebonden Onderzoek) on January 30th 2025 (study number NL87859.099.24) and has been registered in the Centrale Commissie Mensgebonden Onderzoek (CCMO) Research Portal (NL-OMON57433). The research will be conducted in accordance with the principles outlined in the Declaration of Helsinki (latest version available at www.wma.net), the Medical Research Involving Human Subjects Act (WMO), and applicable guidelines, regulations and laws.

### Informed consent

Given the urgency of the condition and the resultant relatively limited time to decide regarding study participation: the patient will be asked to participated after a one-page summary of the patient information has been presented and explained to the patient in the ED (see electronic supplement 1 for the translated version). If the patient provides initial consent to participate, the block is placed and thereafter the full patient information form will be provided, and the decision to participate will be re-evaluated when the patient has had time to read through the full patient information. This approach facilitates quick block placement whilst at the same time offering the patient time to reflect on their decision.

### Study endpoints

#### Primary endpoint.

Primary endpoint is the QoR-15 score (appendix I) 6–8 hours after block placement (or pre-OR, whatever comes first) [23,24]. QoR-15 isa validated patient-related outcome measure (PROM) of postoperative recovery, including questions regarding general well-being with scores ranging from 0 (fully disagree) to 10 (fully agree). The total QoR-score ranges from 0 (extremely poor) to 150 (excellent) [17]. Previous studies have confirmed generalisability and applicability of the QoR-15 in many perioperative settings [17], as well as a good convergent validity between the QoR-15 and pain scores as measured with the VAS [17]. QoR-15 shows excellent internal consistency, split-half reliability, test–retest reliability, and responsiveness [17].

#### Secondary endpoints.

The secondary endpoints of this study are the pre-operative pain scores before block placement,15 min, 30 min, and 1 hour post block placement, block failure (an NRS decrease of less than 2 points between block placement and 1 hour post placement), the amount of opioids used (presented in morphine equivalent dose in mg/hour IV (in the ED) or SC/PO (in the ward) during the first 12 hours after block placement (or until operation), the use of other analgesic medication (for opioids in morphine milligram equivalents) adjusted for exposure time and the number and type of adverse and serious adverse events.

### Data management plan

All data collection is compliant to the Dutch Personal Data Protection Act. All data will be pseudonymized, and a subject identification code list is used to link the data to the subject. The key to the code is safeguarded by the investigator(s). The code is a random number and will not contain any patient identifiable data. All data are stored in encrypted and password protected files on the research drive of the principal investigator in the Frisius MC network. Research data will be stored for 15 years starting from the end of the study.

### Safety considerations

All (serious) adverse events reported by the subject or observed by the investigator or staff will be recorded. Potential SAE’s will be reported to the authorities as mandated by Dutch law through a dedicated Portal As the anticipated number of SAE’s due to the underlying condition (hip fracture) is high, SAE’s that are directly relatable to hip fractures will be exempt from reporting

For all three study procedures (PENG, FICB, FNB) the same local anaesthetics will be used. Potential (S)AEs related to the use of local anaesthetics are represented in Table 2.

**Table 2:** Potential (S)AEs related to the use of local anaesthetics.

AE	SAE
Soreness at the injection site	Local Anaesthetic Systemic Toxicity (LAST)
Tingling feeling	Bleeding from accidental femoral artery puncture
Ringing sound in the ears	Nerve injury
Headache, dizziness, blurred vision and/or confusion	Anaphylaxis
Twitching muscles or shivering	Cardiac arrest
	Any arrhythmia
	Hypotension (systolic blood pressure < 90 mmHg or a drop of >20% compared to baseline)

In case of SAE’s due to systemic toxic effect of the used anaesthetics, or the even more unlikely case of an anaphylactic reaction to ultrasound gel or local anaesthetic, the protocol advises to refer to the hospital’s Local Anaesthetic Systemic Toxicity (LAST) and Anaphylaxis protocols.

### Statistical analysis plan

Standard descriptive statistics will be used to summarize patient characteristics. Continuous data will be evaluated for differences between groups with the One-Way ANOVA (if normally distributed) or Kruskal-Wallis (if not normally distributed). Categorical data will be evaluated for difference between groups using Chi-square testing. Normality of the data will be assessed visually using histograms. Intention to treat analysis will be performed to detect differences in primary outcome (QoR-15) scores between intervention groups will be assessed pairwise with an independent t-test or Mann-Whitney U test, as appropriate. If needed, linear regression will be used to correct for baseline characteristics significantly differing between intervention groups.

Regarding the secondary endpoints, pre-operative pain scores will be examined using a linear mixed-effects model with a random intercept, to assess the impact of nerve blocks on pain over time. The amount of opioid use (expressed in cumulative morphine milligram equivalents), other analgesic medications and the number and type of adverse and serious adverse events will be examined pairwise between intervention groups using the independent t-test, Mann-Whitney U test, or Chi-square test, as appropriate.

An exploratory analysis will assess the impact of the type of LA, the type of fracture (pertrochanteric or neck of femur) and the patient’s age on the effect of the various nerve block types. Linear regression analysis will be performed with an interaction term between the type of block and the type of LA/fracture. If there are significant differences in the outcomes based on the type of local anaesthetic (LA) used or the type of fracture, the results will be stratified accordingly.

Regarding missing data, patient characteristics will be compared between cases with and without complete data. If the percentage of missing data for the primary outcome exceeds 15%, multiple imputation will be considered.

All analyses will be performed according to the intention-to-treat principle. A two-tailed P-value <0.05 will be taken to indicate statistical significance.

### Status and timeline of the study

Participant recruitment and data collection have started on November 7^th^ 2025. It is expected that data collection will take approximately 12 months.

### Patient and public involvement

A focus group discussion with patients who had sustained a hip fracture was organized prior to this study. Patients were consulted about their experiences throughout their whole journey from ED admission to rehabilitation, and about their ideas about dissemination- and implementation of the study findings. The results of this focus group discussion helped us shape the design of the study, and to plan a valorisation approach.

## Discussion

### Strengths and limitations

Literature directly comparing the three different regional anaesthesia options for patients with a hip fracture is scarce and not focussed on early administration of the blocks in the ED before surgery [16]. Additionally, current research does not factor in patient-reported outcomes but is mainly focussed on pain scores [16]. Although pain is an important determinant of wellbeing in the preoperative period, other factors (such as opioid related nausea and vomiting, anxiety, and (in)dependency) play a role as well [4,5,7–9]. Therefore, measuring patient reported outcome measures including these factors is important when different analgesic techniques are evaluated.

We aim to address these knowledge gaps by designing a multi-centre randomized controlled trial to evaluate safety and efficacy of 3 different techniques for regional anaesthesia in patients with hip fractures presenting in the ED. By using a superiority design, we will be able to demonstrate which of these blocks is most effective in improving wellbeing in the time from ED presentation to operation. To measure wellbeing, we use a patient reported outcome measure, the QoR-15 scale, which is validated in various pre-and postoperative settings. In addition, we measure pain scores at various time intervals to allow comparison with previous literature and to monitor the quality of block placement.

The study setting reflects clinical practice, to maximize generalizability of our findings and to facilitate valorisation at a later stage: first, five different hospitals (both university- and regional hospitals) in the Netherlands will participate, each with a unique patient population. Second, blocks will be provided by trained, but non-expert providers (who in general have experience with ultrasound-guided procedures). Third, both patients with neck of femur and pertrochanteric femur fractures are included, as different blocks may prove to be superior for different fracture types, and finally, although equivalent dosages are administered, the choice of local anaesthetic is left to the hospital and not prescribed by the study protocol (with a pre-specified subgroup analysis). By allowing this choice of local anaesthetic to the participating hospital we increase generalizability of our findings. This will not be a confounder on our main outcome measure, as randomisation with a 1:1:1 ratio per centre and stratifying our randomization for the participating centres ensures adequate balancing of the types of LA used over the three different blocks

Another strong point of our study is that the thorough training and briefing of the participating physicians and the development of a dedicated and easy-to-use eCRF to collect study data ensures high-quality data collection, and adherence to the study protocol. Also, pain scores will be registered by ED nurses not involved in the study team to minimize the potential for measurement bias.

Our study design also has a limitation: despite the comprehensive training program to ensure proficiency, experience of participating physicians with the three different blocks may vary, which may affect block placement. We expect to be able to address this by measuring pain scores 15 and 30 minutes after block placement, as an indirect measure of adequacy of block placement.

Thus, despite this limitation, the comprehensive design and systematic protocol of this study are expected to minimize bias and variability, allowing for reliable findings that will contribute significantly to the field of regional anaesthesia for hip fracture patients in the ED.

## Supporting information

S1 File Ethical approval RTPO. (PDF)

S2 File Ethical approval RTPO English translation. (DOCX)

S3 File HSR checklist. (DOCX)

S4 Table SPIRIT checklist. (DOCX)

S5 File QoR-15 questionnaire. (DOCX)

S6 File Protocol that was approved by the ethics committee. (DOCX)

## References

[1] Cooper C, Campion G, Melton LJ 3rd. Hip fractures in the elderly: a world-wide projection. Osteoporos Int. 1992;2(6):285–9. doi: 10.1007/BF01623184 1421796

[2] Medin E, Goude F, Melberg HO, Tediosi F, Belicza E, Peltola M, et al. European regional differences in all-cause mortality and length of stay for patients with hip fracture. Health Econ. 2015;24 Suppl 2:53–64. doi: 10.1002/hec.3278 26633868

[3] Sterling RS. Gender and race/ethnicity differences in hip fracture incidence, morbidity, mortality, and function. Clin Orthop Relat Res. 2011;469(7):1913–8. doi: 10.1007/s11999-010-1736-3 21161737 PMC3111795

[4] Boddaert J, Raux M, Khiami F, Riou B. Perioperative management of elderly patients with hip fracture. Anesthesiology. 2014;121(6):1336–41. doi: 10.1097/ALN.0000000000000478 25299743

[5] Dizdarevic A, Farah F, Ding J, Shah S, Bryan A, Kahn M, et al. A comprehensive review of analgesia and pain modalities in hip fracture pathogenesis. Curr Pain Headache Rep. 2019;23(10):72. doi: 10.1007/s11916-019-0814-9 31388846

[6] Beaudoin FL, Haran JP, Liebmann O. A comparison of ultrasound-guided three-in-one femoral nerve block versus parenteral opioids alone for analgesia in emergency department patients with hip fractures: a randomized controlled trial. Acad Emerg Med. 2013;20(6):584–91. doi: 10.1111/acem.12154 23758305

[7] Bonnet M-P, Mignon A, Mazoit J-X, Ozier Y, Marret E. Analgesic efficacy and adverse effects of epidural morphine compared to parenteral opioids after elective caesarean section: a systematic review. Eur J Pain. 2010;14(9):894.e1-9. doi: 10.1016/j.ejpain.2010.03.003 20381390

[8] Jones JS, Johnson K, McNinch M. Age as a risk factor for inadequate emergency department analgesia. Am J Emerg Med. 1996;14:157–60. 8924137 10.1016/S0735-6757(96)90123-0

[9] Degenhardt L, Grebely J, Stone J, Hickman M, Vickerman P, Marshall BDL, et al. Global patterns of opioid use and dependence: harms to populations, interventions, and future action. Lancet. 2019;394(10208):1560–79. doi: 10.1016/S0140-6736(19)32229-9 31657732 PMC7068135

[10] Braithwaite RS, Col NF, Wong JB. Estimating hip fracture morbidity, mortality and costs. J Am Geriatr Soc. 2003;51:364–70. 12588580 10.1046/j.1532-5415.2003.51110.x

[11] Rashiq S, Vandermeer B, Abou-Setta AM, Beaupre LA, Jones CA, Dryden DM. Efficacy of supplemental peripheral nerve blockade for hip fracture surgery: multiple treatment comparison. Can J Anaesth. 2013;60(3):230–43. doi: 10.1007/s12630-012-9880-8 23334780

[12] Morrison RS, Dickman E, Hwang U, Akhtar S, Ferguson T, Huang J, et al. Regional nerve blocks improve pain and functional outcomes in hip fracture: a randomized controlled trial. J Am Geriatr Soc. 2016;64(12):2433–9. doi: 10.1111/jgs.14386 27787895 PMC5173407

[13] Qiu C, Chan PH, Zohman GL, Prentice HA, Hunt JJ, LaPlace DC, et al. Impact of anesthesia on hospital mortality and morbidities in geriatric patients following emergency hip fracture surgery. J Orthop Trauma. 2018;32(3):116–23. doi: 10.1097/BOT.0000000000001035 29461445

[14] Griffiths R, Babu S, Dixon P, Freeman N, Hurford D, Kelleher E, et al. Guideline for the management of hip fractures 2020: guideline by the association of anaesthetists. Anaesthesia. 2021;76(2):225–37. doi: 10.1111/anae.15291 33289066

[15] Royal College of Emergency Medicine. Best practice guideline - management of pain in adults. 2021.

[16] Dolstra J, Vlieg H, Haak SL, Ter Avest E, Boerma EC, Lameijer H. PENG, fascia-iliaca compartment block or femoral nerve block for pain management of patients with hip fractures. Am J Emerg Med. 2025;96:15–24. doi: 10.1016/j.ajem.2025.06.009 40513549

[17] Myles PS, Shulman MA, Reilly J, Kasza J, Romero L. Measurement of quality of recovery after surgery using the 15-item quality of recovery scale: a systematic review and meta-analysis. Br J Anaesth. 2022;128(6):1029–39. doi: 10.1016/j.bja.2022.03.009 35430086

[18] Aygun H, Tulgar S, Yigit Y, Tasdemir A, Kurt C, Genc C, et al. Effect of ultrasound-guided pericapsular nerve group (PENG) block on pain during patient positioning for central nervous blockade in hip surgery: a randomized controlled trial. BMC Anesthesiol. 2023;23(1):316. doi: 10.1186/s12871-023-02245-3 37715173 PMC10503118

[19] Chen L, Liu S, Cao Y, Yan L, Shen Y. Effect of perioperative ultrasound guided fascia iliaca compartment block in elderly adults with hip fractures undergoing arthroplasty in spinal anesthesia-a randomized controlled trial. BMC Geriatr. 2023;23(1):66. doi: 10.1186/s12877-023-03786-5 36732687 PMC9893664

[20] Nysora. The hip (PENG) block. 2020. https://www.nysora.com/news/the-hip-block-new-addition-to-nysoras-web-app/

[21] Nysora. Ultrasound-guided fascia iliaca nerve block. 2018. https://www.nysora.com/topics/regional-anesthesia-for-specific-surgical-procedures/lower-extremity-regional-anesthesia-for-specific-surgical-procedures/ultrasound-guided-fascia-iliaca-block/

[22] Nysora. Ultrasound-Guided femoral nerve block. 2018. https://www.nysora.com/techniques/lower-extremity/ultrasound-guided-femoral-nerve-block/

[23] Chaudhary K, Bose N, Tanna D, Chandnani A. Ultrasound-guided pericapsular nerve group (PENG) block versus femoral nerve block for positioning during spinal anaesthesia in proximal femur fractures: a randomised comparative study. Indian J Anaesth. 2023;67(10):913–9. doi: 10.4103/ija.ija_553_23 38044928 PMC10691599

[24] Shankar K. Comparative study of ultrasound guided PENG [Pericapsular Nerve Group] block and FIB [Fascia Iliaca Block] for positioning and postoperative analgesia prior to spinal anaesthesia for hip surgeries: prospective randomized comparative clinical study. Indian J Anesth Analg. 2020;7(3):798–803. doi: 10.21088/ijaa.2349.8471.7320.22

