# Correction: Protocol of a multi-centre randomized controlled trial to compare pericapsular nerve group block, fascia-iliaca compartment block and femoral nerve block for pain management in patients with a hip fracture in the emergency department (CPFF-ED)

**DOI:** 10.1371/journal.pone.0344680

**Published:** 2026-03-10

**Authors:** The PLOS One Staff

There is an error in the citation of the fifth author’s name. The correct citation is: Dolstra J, Haak SL, Boerma EC, Lameijer H, Ter Avest E (2026) Protocol of a multi-centre randomized controlled trial to compare pericapsular nerve group block, fascia-iliaca compartment block and femoral nerve block for pain management in patients with a hip fracture in the emergency department (CPFF-ED). PLoS One 21(2): e0342422. https://doi.org/10.1371/journal.pone.0342422.

There are errors in the author affiliations. The correct affiliations are as follows:

Jurian Dolstra^1,2^, Svenja L. Haak^1^, E. Christiaan Boerma^2,3^, Heleen Lameijer^1,3^, Ewoud Ter Avest^4,5^

**1** Department of Emergency Medicine, Frisius Medical Centre Leeuwarden, Leeuwarden, The Netherlands, **2** Department of Sustainable Health, University of Groningen, Campus Fryslân Leeuwarden, Leeuwarden, The Netherlands, **3** Department of Intensive Care, Frisius Medical Centre Leeuwarden, Leeuwarden, The Netherlands, **4** London’s Air Ambulance, London, United Kingdom, **5** Department of Acute Care, University of Groningen, University Medical Centre Groningen, Groningen, The Netherlands.

The publisher apologizes for the errors.
