# Supplementary material for: Protocol of a multi-centre randomized controlled trial to compare pericapsular nerve group block, fascia-iliaca compartment block and femoral nerve block for pain management in patients with a hip fracture in the emergency department (CPFF-ED)
Source: PLoS One. 2026 Feb 9;21(2):e0342422. doi: 10.1371/journal.pone.0342422 (PMC12885299; doi:10.1371/journal.pone.0342422)
Supplement: S1 File — (PDF) [file pone.0342422.s001.pdf]

Regionale Toetsingscommissie
Patiëntgebonden Onderzoek
RTPO
Henri Dunantweg 2
Postbus 888
8901 BR Leeuwarden
Tel. (058) 286 66 66
Doorkiesnr: (058) 286 11 51
Fax (058) 286 79 99
E-mail : RTPO@mcl.nl
www.rtpo.nl
Kamer van Koophandel
Leeuwarden 01079054

J. Dolstra
Promovendus SEH
Frisius MC

Leeuwarden, 30 januari 2025

BESLUIT

Primaire beoordeling.

**NL nummer:**	**NL87859.099.24**	**METC nr.**	**RTPO 1190**
**titel onderzoek:**	**"A comparison of pericapsular nerve group block, Fascia-iliaca compartment block and femoral nerve block for pain management in patients with hip fracture in the emergency department - A randomized controlled trial"**		

Verrichter: Frisius MC, locatie Leeuwarden (voorheen: MCL)

Besluit

De medisch-ethische toetsingscommissie Regionale Toetsingscommissie Patiëntgebonden Onderzoek (RTPO) heeft zich, op grond van artikel 2, tweede lid, aanhef en onder a van de Wet medisch wetenschappelijk onderzoek met mensen (WMO), beraden over bovenstaand onderzoeksdossier.

De commissie **oordeelt positief** over het onderzoeksdossier uit te voeren in het volgende centrum: Frisius MC, locatie Leeuwarden (hoofdonderzoeker Heleen Lameijer).

Dit besluit verliest zijn geldigheid als de inclusie van de eerste proefpersoon niet heeft plaatsgevonden binnen twee jaar na de datum van dit besluit.

Documenten

Het besluit is gebaseerd op de documenten die in bijlage 1 staan vermeld.

Achtergrond

Op 16 oktober 2024 is het onderzoeksdossier ter beoordeling bij de commissie ingediend. Het onderzoeksdossier is besproken in de vergadering van donderdag 31 oktober 2024 en op donderdag 19 december 2024 is de herindiening van 5 december 2024 besproken. De commissie heeft het dagelijks bestuur gemandateerd de reactie en de herziene documenten te beoordelen, welke op 14 januari 2025 zijn ontvangen. Dit heeft plaatsgevonden in het overleg van dinsdag 28 januari 2025.

Overwegingen
De commissie is van oordeel dat aan de voorwaarden in artikel 3, eerste lid, onder a t/m m van de WMO is voldaan. De opmerkingen betroffen voornamelijk de uitvoer en bekwaamheid ten aanzien van de verschillende methoden van behandeling en de patiënteninformatie en zijn nu naar tevredenheid van de commissie aangepast dan wel beantwoordt.

De commissie heeft de in bijlage 1 vermelde onderzoeksverklaringen bekeken. Zij heeft geconstateerd dat is voldaan aan de voorwaarden in artikel 3, eerste lid onder f van de WMO.

De commissie is van oordeel dat het onderzoeksprotocol in een toestemmingsprocedure voorziet die overeenstemt met artikel 6, eerste en tweede lid, van de WMO.

De commissie is van mening dat is voldaan aan de voorwaarden in artikel 6, vijfde t/m negende lid, van de WMO. De proefpersonen worden op gepaste, volledige en begrijpelijke wijze schriftelijk over het onderzoek geïnformeerd en over de mogelijkheid om de toestemming te allen tijde in te trekken.

Verzekeringen
De commissie is van oordeel dat voor de plicht tot het afsluiten van een WMO-proefpersonenverzekering op grond van artikel 7, vijfde lid, van de WMO ontheffing kan worden verleend. Gelet op de aard van het onderzoek lopen de deelnemende proefpersonen naar het oordeel van de commissie geen onderzoeksgerelateerde risico's.

De commissie heeft geconstateerd dat een aansprakelijkheidsverzekering is afgesloten zoals bepaald in artikel 7, negende lid, van de WMO.

Ten slotte wijst de commissie u op de voorwaarden en verplichtingen die in bijlage 2 zijn vermeld.

Hoogachtend,
namens de Regionale Toetsingscommissie Patiëntgebonden Onderzoek,

dr. N.J.G.M. Veeger
voorzitter

Beroepsprocedure
Tegen dit besluit kan een belanghebbende op grond van artikel 23 van de WMO binnen zes weken na de dag waarop het besluit is bekend gemaakt, administratief beroep instellen bij de Centrale Commissie Mensgebonden Onderzoek (CCMO). Het beroepschrift dient u te adresseren aan CCMO, Postbus 16302, 2500 BH Den Haag.

Bijlage 1

Documenten
A1. Aanbiedingsmails 16 oktober 2024;
A1. Correspondentie: vraagbrief RTPO 31 oktober 2024, antwoordmail indiener 5 december 2024, vraagbrief RTPO 23 december 2024, antwoordmail indiener 14 januari 2025;
B1. ABR-formulier versie 03, 13 januari 2025;
C1. Onderzoeksprotocol versie 2, 5 december 2024;
E1/2. Schriftelijke informatie voor de proefpersonen, inclusief bijbehorende toestemmingsverklaring– versie 3, 13 januari 2025;
E4. Simpele versie voorlichtingsmateriaal, versie 1, 13 januari 2025;
F1. Appendix III - QoR-15 vragenlijst
G2. Bewijs dekking aansprakelijkheid van Medisch Centrum Leeuwarden B.V.: polisnummer AB 1000044 van O.W.M. MediRisk B.A., 21 december 2023;
H1. CV H2. CV J. Dolstra – 15-09-2024;
H2. CV H. Lameijer – 19-12-2023;
H3. CV-T. Boeije 04-12-2024;
K3. Concept onderzoekscontract versie 1, 16 oktober 2024;
K6. Note to file statistische analyse, 10 januari 2025.

Bijlage 2

Voorwaarden en verplichtingen

Geldigheid oordeel
Het positieve oordeel verliest zijn geldigheid als de inclusie van de eerste proefpersoon niet heeft plaatsgevonden binnen twee jaar nadat dit besluit is genomen.

Amendementen
Amendementen dienen ter beoordeling aan de RTPO te worden voorgelegd.

Startdatum onderzoek
De RTPO dient op de hoogte te worden gesteld van de definitieve startdatum van het onderzoek. Dat is de datum waarop de inclusie van de eerste proefpersoon plaatsvindt.

Voortgangsrapportage
Eén jaar na datum van het oordeel, en ieder jaar daaropvolgend, dient de RTPO op de hoogte te worden gebracht van de voortgang van de studie middels het formulier Voortgangsrapportage.

Geldigheid verzekering
In het geval het verzekeringscertificaat tijdens de voortgang van het onderzoek zijn geldigheid verliest, dient aan de RTPO tijdig een afschrift van een nieuw geldig certificaat te worden toegestuurd.

Melding SAE's
SAE's dienen aan de RTPO te worden gemeld.

Melding (voortijdige) beëindiging
(Voortijdige) beëindiging van het onderzoek dient, met redenen omkleed, te worden gemeld aan de RTPO.

Eindrapportage
De RTPO dient op de hoogte te worden gebracht van de resultaten van het onderzoek middels een eindrapport.

Termijnen en overige uitleg ten aanzien van de indiening van de verschillende documenten aan de RTPO vindt u op de website van de CCMO.
