# Supplementary material for: Protocol of a multi-centre randomized controlled trial to compare pericapsular nerve group block, fascia-iliaca compartment block and femoral nerve block for pain management in patients with a hip fracture in the emergency department (CPFF-ED)
Source: PLoS One. 2026 Feb 9;21(2):e0342422. doi: 10.1371/journal.pone.0342422 (PMC12885299; doi:10.1371/journal.pone.0342422)
Supplement: S2 File — (DOCX) [file pone.0342422.s002.docx]

**DECISION**

**Primary assessment.**

| **NL nr:** | **NL87859.099.24** | **METC nr.** | **RTPO 1190** |
| --- | --- | --- | --- |
| **Title of the research** | | **“A comparison of pericapsular nerve group block,**  **Fascia-iliaca compartment block and femoral nerve block for pain management in patients with hip fracture in the emergency department - A randomized controlled trial”** | |

**Performer:** Frisius MC, Leeuwarden location (formerly: MCL)

**­­­­­­­­­­­­­­­­__________________________________________________________________________________**

**Decision**

The medical ethics review committee Regionale Toetsingscommissie Patiëntgebonden Onderzoek (RTPO; English translation: Regional Review Committee Patient-related Research) has, on the basis of article 2, second paragraph, exordium and under a of the Wet medisch wetenschappelijk onderzoek met mensen (WMO; English translation: Act Medical Scientific Research Involving Human Subjects), deliberated on the above research file.

The committee **decides** **positively** on the research to be carried out in the following center:

Frisius MC, Leeuwarden location (principal investigator Heleen Lameijer).

This decision will lose its validity if the inclusion of the first test subject has not taken place within two years after the date of this decision.

**Documents**

The decision is based on the documents listed in appendix 1.

**Background**

On 16 October 2024, the research file was submitted to the committee for assessment. The research file was discussed in the meeting of Thursday 31 October 2024 and on Thursday 19 December 2024, the resubmission of 5 December 2024 was discussed. The committee mandated the executive board to assess the response and revised documents, which were received on 14 January 2025. This took place in the meeting of Tuesday 28 January 2025.

**Considerations**

The committee is of the opinion that the conditions in Article 3, paragraph 1, under a to m of the WMO have been met. The comments mainly concerned the implementation and competence with regard to the various treatment methods and patient information and have now been adjusted or answered to the satisfaction of the committee.

The committee has reviewed the research statements listed in Appendix 1. It has found that the conditions in Article 3, paragraph 1, under f of the WMO have been met.

The committee is of the opinion that the research protocol provides for a consent procedure that complies with Article 6, first and second paragraphs, of the WMO.

The committee is of the opinion that the conditions in Article 6, fifth to ninth paragraphs, of the WMO have been met. The subjects are informed in writing about the research in an appropriate, complete and comprehensible manner and about the possibility to withdraw consent at any time.

**Insurance**

The committee is of the opinion that an exemption can be granted for the obligation to take out a WMO test subject insurance on the basis of article 7, paragraph 5, of the WMO. In view of the nature of the research, the participating test subjects do not run any research-related risks in the opinion of the committee.

The committee has established that a liability insurance has been taken out as stipulated in article 7, paragraph 9, of the WMO.

Finally, the committee would like to draw your attention to the conditions and obligations stated in appendix 2.

Yours sincerely,

on behalf of the Regional Review Committee for Patient-related Research,

dr. N.J.G.M. Veeger

chair

**Appeal procedure**

An interested party may lodge an administrative appeal against this decision with the Central Committee on Research Involving Human Subjects (CCMO) on the basis of Article 23 of the WMO within six weeks after the date on which the decision was announced. The notice of appeal should be addressed to CCMO, Postbus 16302, 2500 BH Den Haag.

Appendix 1

**Documents**

**A1.** Offer emails 16 October 2024;

**A1.** Correspondence: RTPO inquiry letter 31 October 2024, submitter's reply email 5 December 2024, RTPO inquiry letter 23 December 2024, submitter's reply email 14 January 2025;

**B1.** ABR-form version 03, 13 January 2025;

**C1.** Research protocol version 2, 5 December 2024;

**E1/2**. Written information for the subjects, including accompanying consent form– version 3, 13 January 2025;

**E4.** Simple version instruction material, version 1, 13 January 2025;

**F1.** Appendix III - QoR-15 questionnaire

**G2.** Proof of liability coverage of Medisch Centrum Leeuwarden B.V.: polisnummer AB 1000044 of O.W.M. MediRisk B.A., 21 December 2023;

**H1.** CV H2. CV J. Dolstra – 15-09-2024;

**H2.** CV H. Lameijer – 19-12-2023;

**H3**. CV-T. Boeije 04-12-2024;

**K3.** Concept research contract version 1, 16 October 2024;

**K6.** Note to file statistical analysis, 10 January 2025.

Appendix 2

Conditions and obligations

**Validity of the decision**

The positive decision will lose its validity if the inclusion of the first subject has not taken place within two years of the decision.

**Amendments**

Amendments must be submitted to the RTPO for assessment.

**Start date of the study**

The RTPO must be informed of the definitive start date of the study. This is the date on which the inclusion of the first subject takes place.

**Progress reporting**

One year after the date of the decision, and annually thereafter, the RTPO must be informed of the progress of the study via the progress reporting form.

**Validity of the insurance**

If the insurance certificate loses its validity during the progress of the study, a copy of a new valid certificate must be sent to the RTPO in a timely manner.

**Notification of SAEs**

SAEs must be reported to the RTPO.

**Notification of (premature) termination**

(Premature) termination of the investigation must be reported to the RTPO with reasons.

**Final report**

The RTPO must be informed of the results of the investigation by means of a final report.

*Deadlines and other explanations regarding the submission of the various documents to the RTPO can be found on the CCMO website.*
