# Supplementary material for: Protocol of a multi-centre randomized controlled trial to compare pericapsular nerve group block, fascia-iliaca compartment block and femoral nerve block for pain management in patients with a hip fracture in the emergency department (CPFF-ED)
Source: PLoS One. 2026 Feb 9;21(2):e0342422. doi: 10.1371/journal.pone.0342422 (PMC12885299; doi:10.1371/journal.pone.0342422)
Supplement: S5 File — (DOCX) [file pone.0342422.s005.docx]

# QoR-15 Vragenlijst – meegeven naar afdeling;

# Afname 6-8 uur na block, of voorafgaand aan OK (indien eerder dan 6-8 uur na block)

| **Uw gegevens** |  | |
| --- | --- | --- |
| Geboortedatum:  Datum van invullen:  Tijd van invullen: | _ _ - _ _ - _ _ _ _  _ _ - _ _ - _ _ _ _  _ _ : _ _ uur |  |
| Randomisatienummer:  Aantal uren sinds plaatsing block: | ________(bijv. 1003)  _________________ |  |

Zie ommezijde

#
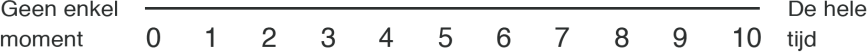
Nederlandstalige QoR-15 vragenlijst

Wilt u het cijfer omcirkelen dat het meest op u van toepassing is?


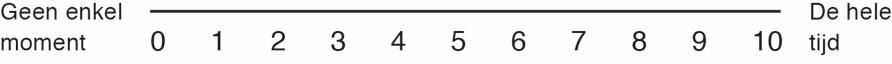

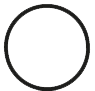


Voorbeeld:

Ik kon makkelijk uit bed stappen

(Mag u het gevraagde niet doen, kies dan 0)

**DEEL A**

**Hoe voelde u zich de afgelopen 24 uur?**

(0 tot 10, waarbij 0 = geen enkel moment [slecht] en 10 = de hele tijd [uitstekend])


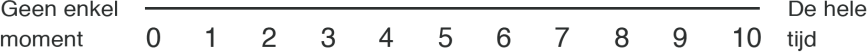

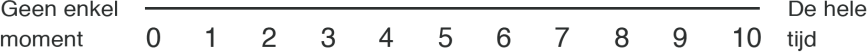

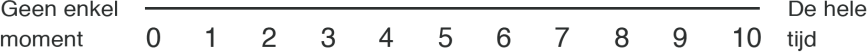
1. Ik kon makkelijk ademhalen

Niet van toepassing


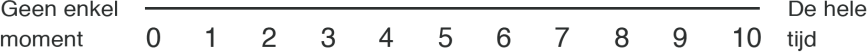


2. Ik kon van eten genieten

3. Ik voelde me uitgerust

n.a.

n.a.


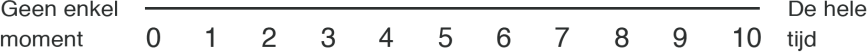


4. Ik heb goed geslapen

5. Ik kon mezelf zonder hulp wassen en verzorgen

n.a.

n.a.


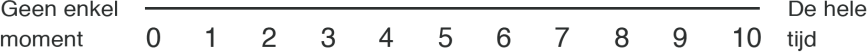


6. Ik kon communiceren met familie of vrienden

7. Ik voelde me gesteund door de artsen en verpleegkundigen in het ziekenhuis

n.a.


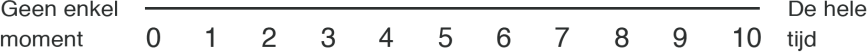


8. Ik kon weer aan het werk of gebruikelijke huishoudelijke activiteiten verrichten

9. Ik voelde me prettig en had het gevoel dat ik zelf kon bepalen wat er gebeurde

n.a.

n.a.


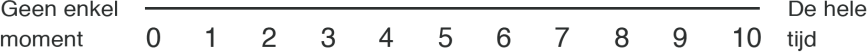


10. Ik voelde me over het algemeen goed

**
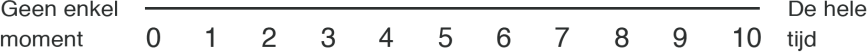
DEEL B**

**In hoeverre heeft u de afgelopen 24 uur last gehad van onderstaande klachten?**

(10 tot 0, waarbij 10 = geen enkel moment [**uitstekend**] en 0 = de hele tijd [slecht])


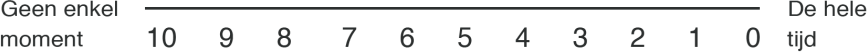

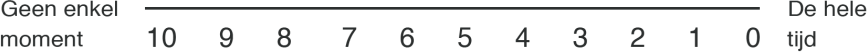
11. Matige pijn


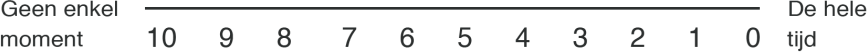


12. Hevige pijn

13. Misselijkheid of braken


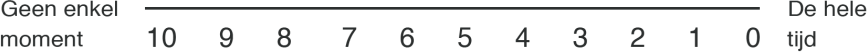


14. Bezorgdheid of angst

15. Verdrietig of somber gevoel


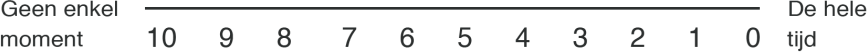
Controleer alstublieft of u **alle** 15 vragen heeft beantwoord. Bedankt voor uw medewerking!
