# Supplementary material for: Protocol of a multi-centre randomized controlled trial to compare pericapsular nerve group block, fascia-iliaca compartment block and femoral nerve block for pain management in patients with a hip fracture in the emergency department (CPFF-ED)
Source: PLoS One. 2026 Feb 9;21(2):e0342422. doi: 10.1371/journal.pone.0342422 (PMC12885299; doi:10.1371/journal.pone.0342422)
Supplement: S6 File — (DOCX) [file pone.0342422.s006.docx]

**A comparison of pericapsular nerve group block, fascia-iliaca compartment block and femoral nerve block for pain management in patients with hip fracture in the emergency department - A randomized controlled trial**

**RESEARCH PROTOCOL – December 2024**

**PROTOCOL TITLE**

A comparison of pericapsular nerve group block, fascia-iliaca compartment block and femoral nerve block for pain management in patients with hip fracture in the emergency department - A randomized controlled trial

| **Protocol ID** | **<*include protocol ID given by sponsor or investigator*>** |
| --- | --- |
| **Short title** | **CPFF-ED** |
| **Version** | **2** |
| **Date** | **5 December 2024** |
| **Coordinating investigator/project leader** | ***Jurian Dolstra, MSc*** |
| **Principal investigator(s) (in Dutch: hoofdonderzoeker/ uitvoerder)** | ***Dr. Heleen Lameijer, MD PhD*** |
|  |  |
| **Sponsor (in Dutch: verrichter/opdrachtgever)** | ***Medical Centre Leeuwarden***  ***8901 BR Leeuwarden*** |
|  | [**www.mcl.nl**](http://www.mcl.nl) |
| **Subsidising party** | ***-*** |
| **Independent expert (s)** | **Tom Boeije, MD** |

**TABLE OF CONTENTS**

1. OBJECTIVES 8

2. STUDY DESIGN 9

3. STUDY POPULATION 10

3.1 Population (base) 10

3.2 Inclusion criteria 10

3.3 Exclusion criteria 10

3.4 Sample size calculation 10

4. TREATMENT OF SUBJECTS 11

4.1 Investigational product/treatment 11

4.1.1 Nerve block procedures 11

4.1.2 Medication and dosage 12

4.2 Rescue medication (if applicable) 13

5. METHODS 13

5.1 Study parameters/endpoints 13

5.1.1 Main study parameter/endpoint 13

5.1.2 Secondary study parameters/endpoints (if applicable) 13

5.2 Randomisation, blinding and treatment allocation 13

5.3 Study procedures 13

5.4 Withdrawal of individual subjects 15

5.5 Replacement of individual subjects after withdrawal 15

5.6 Premature termination of the study 15

6. SAFETY REPORTING 15

6.1 Temporary halt for reasons of subject safety 15

6.2 AEs, SAEs and SUSARs 16

6.2.1 Adverse events (AEs) 16

6.2.2 Serious adverse events (SAEs) 16

6.2.3 Suspected Unexpected Serious Adverse Reactions (SUSARs) 17

6.3 Follow-up of adverse events 19

7. STATISTICAL ANALYSIS 20

8. ETHICAL CONSIDERATIONS 20

8.1 Regulation statement 20

8.2 Recruitment and consent 20

8.3 Benefits and risks assessment, group relatedness 20

9. ADMINISTRATIVE ASPECTS, MONITORING AND PUBLICATION 21

9.1 Handling and storage of data and documents 21

9.2 Amendments 21

9.3 Annual progress report 21

9.4 Temporary halt and (prematurely) end of study report 21

10. REFERENCES 22

**LIST OF ABBREVIATIONS AND RELEVANT DEFINITIONS**

| **ED**  **RCT** | **Emergency Department**  **Randomized Controlled Trial** |
| --- | --- |
| **METC**  **ASA**  **QoR**  **NRS**  **VAS** | **Medical research ethics committee (MREC); in Dutch: medisch-ethische toetsingscommissie (METC)**  **American Society of Anesthesiologists**  **Quality of Recovery**  **Numeric Pain Rating scale**  **Visual Analog Scale** |
| **PENG**  **FICB**  **FNB**  **LA**  **OR** | **Pericapsular Nerve Group block**  **Fascia-iliaca Compartment Block**  **Femoral Nerve Block**  **Local Anaesthetic**  **Operating Room** |
| **(S)AE**  **SAR** | **(Serious) Adverse Event**  **Serious Adverse Reaction** |
| **SUSAR**  **LAST** | **Suspected Unexpected Serious Adverse Reaction**  **Local Anaesthetic Systemic Toxicity** |
| **AVG** | **Dutch Act on Implementation of the General Data Protection Regulation; in Dutch: Uitvoeringswet AVG** |
| **WMO** | **Medical Research Involving Human Subjects Act; in Dutch: Wet Medisch-wetenschappelijk Onderzoek met Mensen** |

**SUMMARY**

**Rationale:**

So far, literature directly comparing the different options to provide regional anaesthesia after a hip fracture is scarce, and not focussed on early administration of nerve blocks in the emergency department (ED).

**Objective**:

The aim of this study is to compare the efficacy and safety of preoperatively placed Pericapsular Nerve Group block (PENG), Fascia-Iliaca Compartment Block (FICB) and Femoral Nerve Block (FNB) for patients with hip fractures in the ED.

**Study design:**

This study is a Randomized Controlled Trial (RCT).

**Study population:**

The research population consists of adult patients (18+) with a proximal femur or neck of femur fracture.

**Intervention (if applicable)**:

Participants will receive either FNB, FICB or PENG block.

**Main study parameters/endpoints:**

The main study parameter is patient-reported QoR-15 score.

**Nature and extent of the burden and risks associated with participation, benefit and group relatedness:**

Although PENG, FICB and FNB are overall safe when performed correctly, there are some risks associated with these procedures. These risks include: bleeding (1-2% chance), infection, damage to surrounding structures, (permanent) nerve injury, and intravascular uptake of local anesthetic resulting in systemic toxicity (LAST). In addition, there is a 5-10% chance that the block provides suboptimal analgesia with the need for additional analgesics. Important to note is that PENG, FICB and FNB are all standard care in the ED. However, since all included types of regional anesthesia in the setting of traumatic femur fractures are part of well-established clinical practice, there is no additional risk for the patients participating in this study. **INTRODUCTION AND RATIONALE**

An aging population will result in a growing prevalence of hip fractures.^1,2^ Hip fractures pose an increased risk of mortality, morbidity, and functional impairment.^3^

Adequate perioperative pain reduction is of utmost importance as pain is an important factor influencing complication rates, posing a significant risk for decreased functional outcomes, prolonged time to mobilization and longer hospital stay.^4,5^ Systemic analgesia is commonly used in the pain management of patients with hip fractures, including opioids (e.g. morphine or fentanyl).^6^ However, opioids are associated with adverse events such as delirium, vomiting, pruritus and respiratory depression, which are even more common in older patients.^7,8^ Additionally, opioids are highly addictive, as illustrated by the ongoing worldwide opioid crisis.^9^

An alternative method to obtain adequate analgesia is the use of regional nerve blocks. Nerve blocks have several advantages over systemic analgesia. First, they may provide superior analgesia.^5^ Second, they contribute to a reduction in opioid use and opioid-related adverse events, and thereby decrease the risk of delirium and cognitive dysfunction.^10-12^ Previous research has shown that survival and length of hospital stay are both positively associated with the use of regional anesthesia in hip fractures.^13^ Recent guidelines therefore advise to use regional anaesthetic techniques for pain management in hip fractures.^14-16^

Various different techniques can be applied to perform regional anaesthesia for patients with hip fractures, such as the femoral nerve block (FNB) or the fascia iliaca compartment block (FICB). While FNB and FICB reduce pain in proximal femur fractures, they do not provide adequate analgesia under all circumstances.^17^ As both blocks primarily target the femoral nerve, this may be explained by inadequate blockage of the obturator nerve and/or the lateral femoral cutaneous nerve.^18-25^ More recently, the pericapsular nerve group (PENG) block has been introduced as an alternative. The PENG block, being a more cranially placed block, has the potential to target the obturator nerve better and to thereby provide better overall limb analgesia.^20,26^

So far, literature directly comparing the different options to provide regional anaesthesia after a hip fracture is scarce, and not focussed on early administration of the blocks in the emergency department (ED) before hip fracture surgery.

The aim of this study is therefore to compare the efficacy and safety of preoperatively placed PENG, FICB and FNB by assessing pain, opioid use and patient reported outcomes in patients with hip fractures presented in the ED.

# OBJECTIVES

**Primary Objective:**

To compare patient reported outcomes as measured with the QoR-15 questionnaire between patients with hip (proximal femur) fractures receiving three different pre-operative blocks in the ED: PENG block, Fascia-iliaca compartment Block or Femoral Nerve Block.

**Hypothesis:**

**H0:** There is no significant difference in the QoR-15 scores between patients receiving PENG, FICB or FNB in the ED.

**H1:** There is a significant difference in the QoR-15 scores between patients receiving PENG, FICB or FNB in the ED.

**Secondary Objective(s):**

- To compare pre-operative pain scores 15 min, 30 min, and 1 hour post-block placement in patients receiving PENG, FICB and FNB in the ED.
- To compare the amount of rescue medication, calculated as mg/hour given IV in the ED after block placement or SC/IO in the ward during the first 12 hours after block placement or until operation.
- To compare complications of block placement in patients receiving PENG, FICB and FNB in the ED in the first 24 hours after block placement.

# STUDY DESIGN

This study will be a non-blinded, multicentre Randomized Controlled Trial (RCT), conducted in the ED of the MCL and other cooperating hospitals. Patients with a confirmed proximal- or neck of femur fracture are randomized to one of three treatment arms: FICB, FNB or PENG. After randomization regional anaesthesia is provided according to a standardized protocol and pain scores are measured at fixed time intervals. Patient reported outcomes are measured with a validated (QoR-15) questionnaire either 6-8 hours after block administration or before admittance to the operating room (OR), whichever one comes first. A flowchart of the study design can be found below (*Figure 1*).

**Figure 1**. Flowchart of the study design.

**Study design**

Hip fracture is confirmed in patient via X-ray, indication for nerve block is established and informed consent is obtained.

Patient with presumed hip fracture is presented at the ED.

Pain score and prehospital analgesics administered are obtained.

Randomization to one of three treatment arms.

Nerve Block is administered in the ED by ED clinician

The clinician/nurse continues the care process and registers study data.

*For a detailed figure on data collection, see* [*5.3 study procedures*](#_Study_procedures)*.*

Patient is admitted from ED to designated department elsewhere in the hospital.

At 6-8 hours post-block, or pre-OR (whichever one comes first), additional data are collected.

*For a detailed figure on data collection, see* [*5.3 study procedures*](#_Study_procedures)*.*

# STUDY POPULATION

## Population (base)

The research population will consist of adult patients with a femoral neck fracture or intertrochanteric femur fracture.

## Inclusion criteria

In order to be eligible to participate in this study, a subject must meet all of the following inclusion criteria:

- 18 years or older.
- proximal- or neck of femur fracture requiring surgical intervention.
- Able and willing to provide informed consent and reliably report symptoms to the research team.

## Exclusion criteria

Patients meeting any of the following criteria will be excluded from participation in this study:

- Known allergy to local anaesthetics.
- Infection at the injection-site.
- Periprosthetic fracture.
- Skin injury, local infection or recent burns hindering the use of ultrasound for ultrasound guided nerve block placement.
- Multiple fractures simultaneously.
- Pain score of < 2 (range 0 - 10) or < 20 (range 0 - 100) in rest or when moving in bed, when presented to the ED.

## Sample size calculation

Sample size was based on the primary outcome measure (QoR-15 score) and a superiority design. Following this design, an independent samples t-test was used, reasoning that one block would be superior to all others if it outperforms its best performing competitor. Based on previous research, a clinically relevant difference is determined at 6.0 points difference in QoR-15 scores.^27^ Also based on previous literature, a standard deviation of 10 is used.^28-30^ A power of 0.9 is used to minimize the chance of missing differences between the groups, as the differences in scores are likely to be small. In order to detect this difference between any of the three groups with a pre-defined p-value of 0.05, 180 patients are needed (distributed over three groups of 60). To account for 10% attrition, i.e. potential dropouts during the study process, a total of 198 patients will be included.

# TREATMENT OF SUBJECTS

## Investigational product/treatment

### Nerve block procedures

Patients will be randomized into one of three different groups, depending on which regional nerve block type they will receive for pre-operative pain management. The three regional nerve block types are described as follows:

**Femoral Nerve Block**

The femoral nerve block is performed with the patient in the supine position, and guided by ultrasound. The ultrasound transducer is placed transversely on the inguinal crease, and moved in a lateral-to-medial direction to identify the femoral artery. The needle tip is placed immediately adjacent to the lateral aspect of the femoral nerve, below the fascia iliaca, that surrounds the femoral nerve. Negative aspiration should be observed before injection of local anaesthetic into the plane and observation of fluid spread in the desired plane. Then 15 ml of local anaesthetic is injected. Proper deposition of local anaesthetic is confirmed either by observation of the femoral nerve being displaced by the injectate or by the spread of the local anaesthetic above or below the nerve, surrounding and separating it from the fascia iliaca layers. If the spread is deemed inadequate, injection is stopped and the needle is repositioned more laterally or medially to facilitate adequate block placement.

**Fascia Iliaca Compartment Block**

The fascia iliaca nerve block (also called the fascia iliaca compartment nerve block) is performed with the patient in the supine position, and guided by ultrasound. The femoral artery is visualized by placement of the transducer transversely on the inguinal crease. The goal is to place the needle tip under the fascia iliaca approximately at a lateral third of the line connecting the anterior superior iliac spine to the pubic tubercle. Negative aspiration should be observed before injection of local anaesthetic into the plane and observation of fluid spread in the desired plane. The total volume of the injection should be 40 ml. To achieve this, the dose of local anaesthetic (according to the dosing recommendations in Table 1), is topped up to a volume of 40 ml with NaCL. Its spread laterally toward the iliac spine and medially toward the femoral nerve is observed with US visualization. A proper injection will result in the separation of the fascia iliaca by the local anaesthetic in the medial–lateral direction from the point of injection as described. If the spread is deemed inadequate, injection is stopped and the needle is repositioned more laterally or medially to facilitate adequate block placement.

**Pericapsular Nerve Group block**

The PENG block is performed with the patient in the supine position, and guided by ultrasound. The ultrasound probe is initially placed in a transverse plane over the anterior inferior iliac spine, before being aligned with the pubic ramus by rotating the probe 45 degrees counter clockwise. The iliopubic eminence, the iliopsoas muscle tendon, the femoral artery and pectineus muscle should be visualised in this view. The femoral nerve should be identified during scanning prior to needle insertion. The goal is to place the needle tip in the musculofascial plane between the psoas tendon anteriorly and the pubic ramus posteriorly. The total volume of the injection should be 40 ml. To achieve this, the dose of local anaesthetic (according to the dosing recommendations in Table 1), is topped up to a volume of 40 ml with NaCL. Negative aspiration should be observed before injection into the plane and observation of fluid spread in the desired plane. If the spread is deemed inadequate, injection is stopped and the needle is repositioned more laterally or medially to facilitate adequate block placement.

### Medication and dosage

As allocation to the treatment arms will be balanced for each participating centre, participating medical centres should use the NYSORA procedures for ultrasound guided nerve block placement.^31-33^ The type of LA (such as levobupivacaine or ropivacaine) used can be the one of their preference. While doing so, they have to adhere to the dosing recommendations in Table 1. In line with existing guidelines, high-volume low-concentration blocks are advised for FICB and PENG blocks specifically.

|  | **Levobupivacaine** | **Ropivacaine** |
| --- | --- | --- |
| Relative maximal allowed dosage | 2mg/kg | 3mg/kg |
| Absolute maximal allowed dosage | 200mg | 300mg |
| Advised dosage  Weight of 50-60kg  Weight of >60kg | 75mg  100mg | 112,5mg  150mg |

Table 1. Allowed and advised dosages of local anaesthetics.

## Rescue medication (if applicable)

Cooperating medical centres can use their standard, already existing procedures for escape medication by means of opioids. To account for different opioids used in different centers, dose equivalents will be calculated for comparison.

# METHODS

## Study parameters/endpoints

### Main study parameter/endpoint

Score on the QoR-15 questionnaire.

### Secondary study parameters/endpoints (if applicable)

- - Pre-operative pain scores at 15 min-, 30 min-, and 1 hour post-block placement.
  - The amount of rescue medication calculated as mg/hour given IV in the ED after block placement or SC/IO in the ward during the first 12 hours after block placement or until operation.
  - Number and type of adverse and serious adverse events.

## Randomisation, blinding and treatment allocation

Block-randomization will be performed via the REDCap randomization module on a 1:1:1 basis for every participating centre.

## Study procedures

Before the start of the data collection, a refamiliarization session for all three nerve block procedures will be hosted in each individual center. In this refamiliarization session, an expert in POCUS will provide theoretical and practical training in block placement for each of the three blocks, and will test and log the skill level of the participating physicians in a dedicated training log. Only physicians who are able to perform all three blocks in line with the NYSORA guidelines are eligible for participation in the study.^31-33^


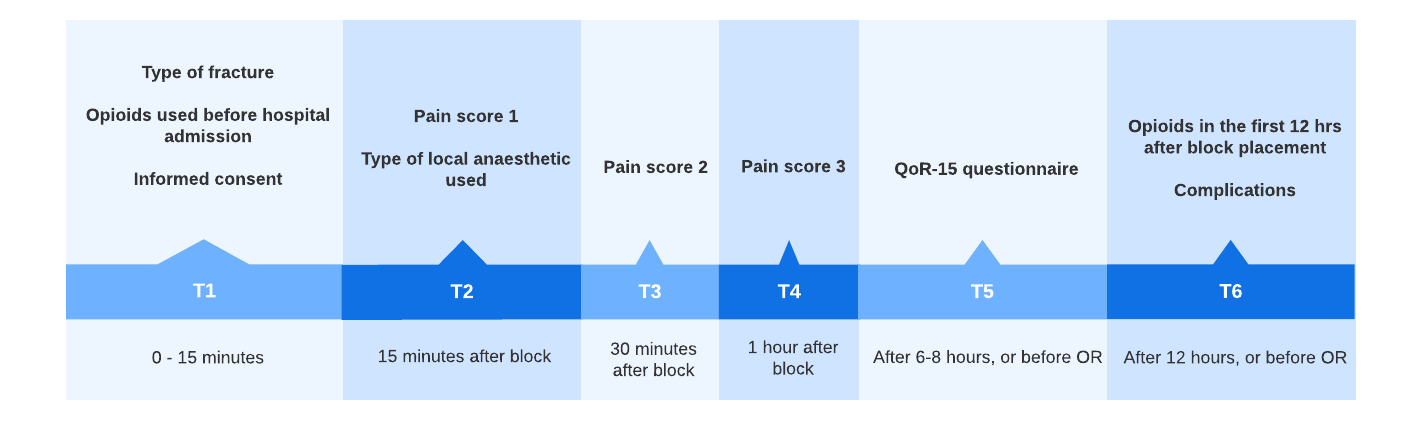


**Figure 2**. Timeline of the study procedure, showing which data will be collected at what time point.

Data will be gathered at 6 different time points (T1-T6). *Figure 2* shows a detailed timeline of data collection.

Pre-block characteristics (demographic data (age, sex, weight), ASA score, pain scores (VAS) during rest and light elevation of the affected leg, type of fracture, analgesics administered before hospital admission (including medication routinely used by the patient), block characteristics (type of block, visualization of landmarks, amount- and type of LA administered, timing of block placement), and post-block characteristics (total amount of opioids administered in the first 12 hours after block administration and (S)AE’s) will be reported in an electronic case report form (appendix IV).

The primary endpoint, quality of recovery, will be measured either 6-8 hours post-block, or pre-OR (whichever one comes first) with the Quality of recovery-15 (QoR) patient-reported outcome questionnaire that measures the quality of recovery after surgery and anaesthesia.^34^ To ensure patients will be adequately rested for their operation, QoR-15 registration will be postponed until the next morning for blocks placed between 17.00 and 22.00 hours, as QoR-15 registration would then fall between 23.00 and 6.00. The QoR-15 questionnaire can be found in the appendices (*Appendix III*). Previous studies have shown a good convergent validity between the QoR-15 and the visual analog scale (VAS). Additionally, validity of the QoR-15 is supported by a negative correlation with duration of surgery and duration of hospital stay, and the QoR-15 shows excellent internal consistency, split-half reliability, test–retest reliability, and responsiveness.^34^

As the QoR-15 questionnaire needs to be collected 6-8 hours after block administration, or before admittance to the OR (whichever one comes first), a member of the research team will visit the patient at their designated department (surgery or orthopaedics) to collect this data when 6-8 hours have passed since block administration, or pre-OR.

## Withdrawal of individual subjects

Subjects can leave the study at any time for any reason if they wish to do so without any consequences. The investigator can decide to withdraw a subject from the study for urgent medical reasons.

## Replacement of individual subjects after withdrawal

Not applicable.

## Premature termination of the study

Can be performed for unforeseen indications as stated under safety reporting.

# SAFETY REPORTING

## Temporary halt for reasons of subject safety

In accordance to section 10, subsection 4, of the WMO, the investigator will suspend the study if there is sufficient ground that continuation of the study will jeopardise subject health or safety. The investigator will notify the accredited METC without undue delay of a temporary halt including the reason for such an action. The study will be suspended pending a further positive decision by the accredited METC. The investigator will take care that all subjects are kept informed.

## AEs, SAEs and SUSARs

### Adverse events (AEs)

Adverse events are defined as any undesirable experience occurring to a subject during the study, whether or not considered related to the intervention. All adverse events reported spontaneously by the subject or observed by the investiga­tor or staff will be recorded. All three of the procedures (PENG, FICB, FNB) are common clinical practice and carry the same side effects related to the use of local anaesthetics.

AE’s related to the use of local anaesthetics are:

- Soreness at the injection site;
- Tingling feeling;
- Ringing in the ears;
- Headache, dizziness, blurred vision and/or confusion;
- Twitching muscles or shivering;

### Serious adverse events (SAEs)

A serious adverse event is any untoward medical occurrence or effect that

- results in death;
- is life threatening (at the time of the event);
- requires hospitalisation or prolongation of existing inpatients’ hospitalisation;
- results in persistent or significant disability or incapacity;
- any other important medical event that did not result in any of the outcomes listed above due to medical or surgical intervention but could have been based upon appropriate judgement by the investigator.

*An elective hospital admission will not be considered as a serious adverse event.*

SAE’s related to the use of local anaesthetics, although very uncommon, are:

- Local Anaesthetic Systemic Toxicity (LAST);
- Bleeding from accidental femoral artery puncture;
- Nerve injury;
- Anaphylaxis;
- Cardiac arrest;
- Any arrhythmia;
- Hypotension (systolic blood pressure < 90 mmHg or a drop of >20% compared to baseline).

In case of SAEs due to systemic toxic effect of the used anaesthetics, physicians are supposed to handle by using their existing local LAST-protocol (Appendix I: example of a LAST protocol). This is exactly the same procedure for all three block types and is therefore not different to the standard way of care. In the even more unlikely case of an anaphylactic reaction to ultrasound gel or local anaesthetic, the hospitals local protocol ‘Anaphylaxis’ (‘Anafylaxie’) will be used (Appendix II: example of an anaphylaxis treatment protocol).

The investigator will document all SAEs, including those related to the underlying condition (hip fracture). An overview of SAEs will be provided in the annual overview report. SAE’s potentially related to the use of LA, from now on named SAR (Serious Adverse Reaction), will be reported as mentioned in paragraph 6.2.3. As the anticipated number of SAE’s due to the underlying condition (hip fracture) is high, SAE’s that are directly relatable to hip fractures will be exempt from reporting to the Toetsingonline portal.

### Suspected Unexpected Serious Adverse Reactions (SUSARs)

An SAE that occurs during research with an investigational product (LA in this study) may be a SAR or a SUSAR (Suspected Unexpected Serious Adverse Reaction).

An SAE is a SAR if there is a certain degree of probability that the SAE is a harmful and undesired reaction to LA, regardless of the administered dose. If the SAR is unexpected it is called a SUSAR. Following documentation of an SAE, the investigator and the independent expert will determine whether the SAE could be related to LA use. Figure 3 provides definitions of relatedness.

In case it is decided that the SAE is related to LA use following the definitions found in Figure 3 (thus being a SUSAR), it will be reported by the investigator through the web portal *ToetsingOnline* to the accredited METC that approved the protocol, within 7 days of first knowledge for SUSARs that result in death or are life threatening followed by a period of maximum of 8 days to complete the initial preliminary report. All other SUSARs will be reported within a period of maximum 15 days after first knowledge of the serious adverse events.


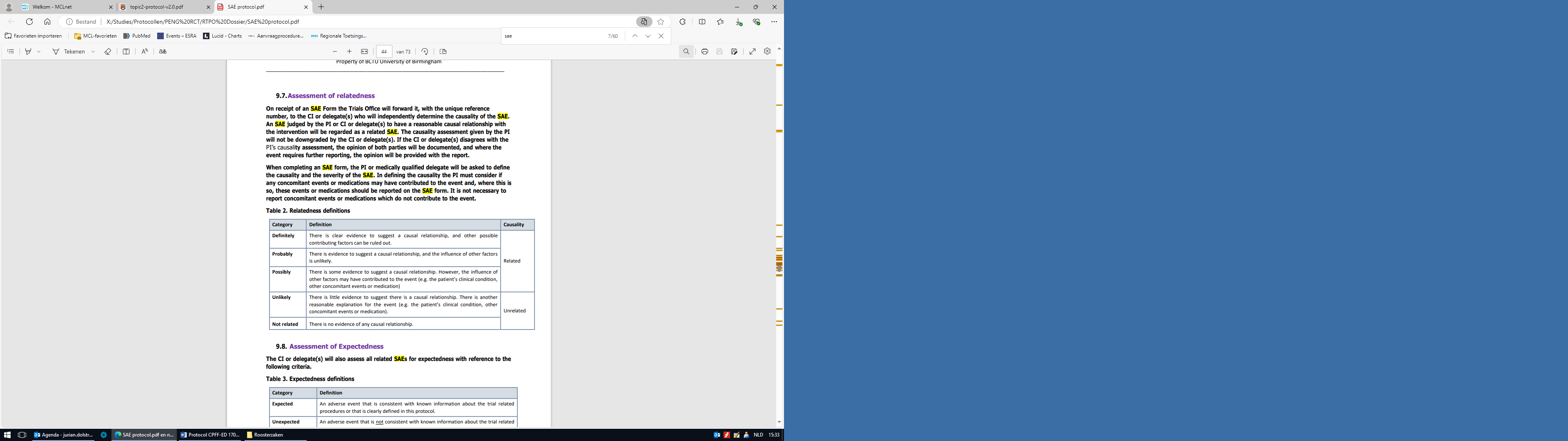
As the anticipated number of SAE’s due to the underlying condition (hip fracture) is high, SAE’s that are directly relatable to hip fractures will be exempt from reporting to the Toetsingonline portal.

**Figure 3**. Definitions of relatedness.

SAE’s are certainly not related to LA use when they occur more than 12 hours after block administration, as the LA used in this study work for 8-12 hours.

## Follow-up of adverse events

All AEs will be followed until they have abated, or until a stable situation has been reached. Depending on the event, follow up may require additional tests or medical procedures as indicated, and/or referral to the general physician or a medical specialist.

SUSARs need to be reported till end of study within the Netherlands, as defined in the protocol.

# STATISTICAL ANALYSIS

Standard descriptive statistics will be used to summarize patient characteristics. Continuous data will be inspected for normality before analysis (Shapiro-Wilk), summarized with mean and standard deviation if normally distributed, or median and 25-75th centiles (Q1, Q3) if not normally distributed, and evaluated for differences with the One-Way ANOVA (if normally distributed) or Friedman Test (if not normally distributed). Categorical data will be summarized by frequency count and percentage calculations. Examination of possible associations between data will be done by Chi-Square Test or Logistic Regression. LA-type will be used as an interaction term to investigate the influence of the type of LA used. If needed, quantile regression, suitable for skewed data, will be used to identify confounders that may impact on postoperative quality of recovery scores. A two-tailed P-value <0.05 will be taken to indicate statistical significance.

# ETHICAL CONSIDERATIONS

## Regulation statement

Permission for conduction of the study and gathering of information in the electronic patient journal will be received from the Ethical Committee of the medical centre Leeuwarden Friesland, the Netherlands.

We specifically seek permission to access all relevant journal entries in the participants electronic journal regarding the current hip fracture and its treatment from the point of primary contact to the ED and for the remainder of the study period.

## Recruitment and consent

Eligible patients will be asked to participate, and will be referred to the patient information form. The purpose of the study and procedure will additionally be explained and there will be room for questions. If the patient would like to participate, written and oral informed consent will be obtained.

## Benefits and risks assessment, group relatedness

There is no increased risk or potential benefit for patients participating in the study, as PENG, FICB and FNB are all standard care and are currently performed in the ED. When any block is found to be superior by this study, this knowledge will however benefit the whole future population of patients with hip fractures.

# ADMINISTRATIVE ASPECTS, MONITORING AND PUBLICATION

## Handling and storage of data and documents

We will use the secure web application REDCap. Data will be collected through an electronic case report form (eCRF) and later digitally archived for a duration of 10 years. Data will be collected and processed by the research team. Only the research team has access to the coded study files. Data will be exported to SPSS to facilitate further analysis.

## Amendments

Amendments are changes made to the research after a favourable opinion by the accredited METC has been given. All amendments will be notified to the METC that gave a favourable opinion.

## Annual progress report

The investigator will submit a summary of the progress of the trial to the accredited METC once a year. Information will be provided on the date of inclusion of the first subject, numbers of subjects included and numbers of subjects that have completed the trial, serious adverse events/ serious adverse reactions, other problems, and amendments.

## Temporary halt and (prematurely) end of study report

The investigator will notify the accredited METC of the end of the study within a period of 8 weeks. The end of the study is defined as the last patient’s last visit.

The investigator will notify the METC immediately of a temporary halt of the study, including the reason of such an action. In case the study is ended prematurely, the investigator will notify the accredited METC within 15 days, including the reasons for the premature termination.

Within one year after the end of the study, the investigator will submit a final study report with the results of the study, including any publications/abstracts of the study, to the accredited METC.

# REFERENCES

1. Cooper C, Campion G, Melton Iii L. Hip fractures in the elderly: A world-wide projection. Osteoporosis Int. 1992;2(6):285-89.
2. Medin E, Goude F, Melberg HO, et al. European regional differences in allcause mortality and length of stay for patients with hip fracture. Health Econ 2015; 24(Suppl 2):53–64.
3. Sterling RS. Gender and race/ethnicity differences in hip fracture incidence, morbidity, mortality, and function. Clin Orthop Relat Res. 2011 Jul;469(7):1913-8. doi: 10.1007/s11999-010-1736-3. PMID: 21161737; PMCID: PMC3111795.
4. Boddaert J, Raux M, Khiami F, Riou B. Perioperative management of elderly patients with hip fracture. Anesthesiology. 2014;121(6):1336–41
5. Dizdarevic A, Farah F, Ding J, et al. A comprehensive review of analgesia and pain modalities in hip fracture pathogenesis. Curr Pain Headache Rep. 2019;23(10):72-9. doi: 10.1007/s11916-019-0814-9.
6. Beaudoin FL, Haran JP, Liebmann O. A comparison of ultrasound-guided three-in-one femoral nerve block versus parenteral opioids alone for analgesia in emergency department patients with hip fractures: A randomized controlled trial. Acad Emerg Med. 2013;20(6):584-591. doi: 10.1111/acem.12154.
7. Bonnet M, Mignon A, Mazoit J, Ozier Y, Marret E. Analgesic efficacy and adverse effects of epidural morphine compared to parenteral opioids after elective caesarean section: A systematic review. Eur J Pain. 2010;14(9):894.e1-894.e9. doi: 10.1016/j.ejpain.2010.03.003.
8. J.S. Jones, K. Johnson, M. McNinch, Age as a risk factor for inadequate emergency

department analgesia, Am. J. Emerg. Med. 14 (1996) 157–160.

1. Degenhardt L, Grebely J, Stone J, Hickman M, Vickerman P, Marshall BDL, Bruneau J, Altice FL, Henderson G, Rahimi-Movaghar A, Larney S. Global patterns of opioid use and dependence: harms to populations, interventions, and future action. Lancet. 2019 Oct 26;394(10208):1560-1579. doi: 10.1016/S0140-6736(19)32229-9. Epub 2019 Oct 23. PMID: 31657732; PMCID: PMC7068135.
2. R.S. Braithwaite, N.F. Col, J.B. Wong, Estimating hip fracture morbidity, mortality

and costs, J. Am. Geriatr. Soc. 51 (2003) 364–370.

1. Rashiq S, Vandermeer B, Abou-Setta AM, Beaupre LA, Jones CA, Dryden DM. Efficacy of supplemental peripheral nerve blockade for hip fracture surgery: multiple treatment comparison. Can J Anesth. 2013;60(3):230–43.
2. Morrison RS, Dickman E, Hwang U, Akhtar S, Ferguson T, Huang J, et al. Regional nerve blocks improve pain and functional outcomes in hip fracture: a randomized controlled trial. J Am Geriatr Soc. 2016;64:2433–9
3. Qiu C, Chan PH, Zohman GL, Prentice HA, Hunt JJ, LaPlace DC, et al. Impact of anesthesia on hospital mortality and morbidity in geriatric patients following emergency hip fracture surgery. J Orthop Trauma. 2018;32(3):116–23
4. Bailey CR, Radhakrishna S, Asanati K, Dill N, Hodgson K, McKeown C, Pawa A, Plaat F, Wilkes A. Ergonomics in the anaesthetic workplace: Guideline from the Association of Anaesthetists. Anaesthesia. 2021 Dec;76(12):1635-1647. doi: 10.1111/anae.15530. Epub 2021 Jul 12. PMID: 34251028; PMCID: PMC9292255.
5. Griffiths R, Babu S, Dixon P, Freeman N, Hurford D, Kelleher E, Moppett I, Ray D, Sahota O, Shields M, White S. Guideline for the management of hip fractures 2020: Guideline by the Association of Anaesthetists. Anaesthesia. 2021 Feb;76(2):225-237. doi: 10.1111/anae.15291. Epub 2020 Dec 2. PMID: 33289066.
6. The Royal College of Emergency Medicine (2021). Best Practice Guideline - Management of Pain in Adults. [RCEM_BPC_Management_of_Pain_in_Adults_300621.pdf](https://rcem.ac.uk/wp-content/uploads/2021/10/RCEM_BPC_Management_of_Pain_in_Adults_300621.pdf)
7. Bonnet M, Mignon A, Mazoit J, Ozier Y, Marret E. Analgesic efficacy and adverse effects of epidural morphine compared to parenteral opioids after elective caesarean section: A systematic review. Eur J Pain. 2010;14(9):894.e1-894.e9. doi: 10.1016/j.ejpain.2010.03.003.
8. Griffiths R, Babu S, Dixon P, Freeman N, Hurford D, Kelleher E, Moppett I, Ray D, Sahota O, Shields M, White S. Guideline for the management of hip fractures 2020: Guideline by the Association of Anaesthetists. Anaesthesia. 2021 Feb;76(2):225-237. doi: 10.1111/anae.15291. Epub 2020 Dec 2. PMID: 33289066.
9. Capdevila X, Biboulet P, Bouregba M, Barthelet Y, Rubenovitch J, d'Athis F. Comparison of the three-in-one and fascia iliaca compartment blocks in adults: Clinical and radiographic analysis. Anesth Analg. 1998;86(5):1039-1044. doi: 10.1097/00000539-199805000-00025.
10. Garip L, Balocco AL, Van Boxstael S. From emergency department to operating room: Interventional analgesia techniques for hip fractures. Curr Opin Anaesthesiol. 2021;34(5):641-647. doi: 10.1097/ACO.0000000000001046.
11. Desmet M, Balocco AL, Van Belleghem V. Fascia iliaca compartment blocks: different techniques and review of the literature. Best Pract Res Clin Anaesthesiol 2019; 33:57–66.
12. Nielsen ND, Greher M, Moriggl B, et al. Spread of injectate around hip articular sensory branches of the femoral nerve in cadavers. Acta Anaesthesiol Scand. 2018;62(7):1001-1006. doi: 10.1111/aas.13122.
13. Bravo D, Layera S, Aliste J, et al. Lumbar plexus block versus suprainguinal fascia iliaca block for total hip arthroplasty: a single-blinded, randomized trial. J Clin Anesth. 2020;66:109907.
14. Qian Y., Guo Z., Huang J., Zhang Q., An X., Hu H., et. al.: Electromyographic comparison of efficacy of ultrasound-guided suprainguinal and infrainguinal fascia iliaca compartment block for blockade of the obturator nerve in total knee arthroplasty: a prospective randomized controlled trial. Clin J Pain 2020; 36: pp. 260-266.
15. Giron-Arango L, Peng PWH, Chin KJ, Brull R, Perlas A. Pericapsular nerve group (PENG) block for hip fracture. Reg Anesth Pain Med. 2018;43(8):859-863. doi: 10.1097/AAP.0000000000000847.
16. Kim JY, Kim J, Kim D, et al. Anatomical and radiological assessments of injectate spread stratified by the volume of the pericapsular nerve group block. Anesth Analg. 2023;136(3):597-604. doi: 10.1213/ANE.0000000000006364.
17. Myles PS, Myles DB. An Updated Minimal Clinically Important Difference for the QoR-15 Scale. Anesthesiology. 2021 Nov 1;135(5):934-935. doi: 10.1097/ALN.0000000000003977. PMID: 34543410.
18. Aygun H, Tulgar S, Yigit Y, Tasdemir A, Kurt C, Genc C, Bilgin S, Senoğlu N, Koksal E. Effect of ultrasound-guided pericapsular nerve group (PENG) block on pain during patient positioning for central nervous blockade in hip surgery: a randomized controlled trial. BMC Anesthesiol. 2023 Sep 15;23(1):316. doi: 10.1186/s12871-023-02245-3. PMID: 37715173; PMCID: PMC10503118.
19. Chen L, Liu S, Cao Y, Yan L, Shen Y. Effect of perioperative ultrasound guided fascia iliaca compartment block in elderly adults with hip fractures undergoing arthroplasty in spinal anesthesia-a randomized controlled trial. BMC Geriatr. 2023 Feb 2;23(1):66. doi: 10.1186/s12877-023-03786-5. PMID: 36732687; PMCID: PMC9893664.
20. Ismail T I, Elshafie M H , Patient-reported outcomes after femoral nerve block versus periarticular injections in patients undergoing total knee arthroplasty: A randomized controlled study. Indian J Clin Anaesth 2020;7(4):687-694
21. Nysora. (2020, February 13). The hip (PENG) block. NYSORA. https://www.nysora.com/news/the-hip-block-new-addition-to-nysoras-web-app/
22. Nysora. (2018, September 21). Ultrasound-Guided Fascia Iliaca Nerve Block. NYSORA. https://www.nysora.com/topics/regional-anesthesia-for-specific-surgical-procedures/lower-extremity-regional-anesthesia-for-specific-surgical-procedures/ultrasound-guided-fascia-iliaca-block/
23. Nysora. (2018, September 21). Ultrasound-Guided femoral nerve block. NYSORA. <https://www.nysora.com/techniques/lower-extremity/ultrasound-guided-femoral-nerve-block/>
24. Myles PS, Shulman MA, Reilly J, Kasza J, Romero L. Measurement of quality of recovery after surgery using the 15-item quality of recovery scale: a systematic review and meta-analysis. Br J Anaesth. 2022 Jun;128(6):1029-1039. doi: 10.1016/j.bja.2022.03.009. Epub 2022 Apr 14. PMID: 35430086.

**Appendix I**

LAST protocol (additional pdf file)

**Appendix II**

Anaphylaxis (‘Anafylaxie’) (additional pdf file)

**Appendix III**

QoR-15 questionnaire in Dutch

**Appendix IV**

Case report form in Dutch

**Appendix V**

Information for patients and Consent form (in Dutch)

Appendix III

**Vragenlijst**

± 6-8 uur na plaatsing block **OF** voor OK

**Uw gegevens**

Datum van invullen: …. - …. - ….

Tijd van invullen: …. : …. uur *(zoals 9:30, op het moment dat het half 10 in de ochtend is)*.

Aantal uren sinds plaatsing van het block: …. : …. uur (*zoals 9:30)*


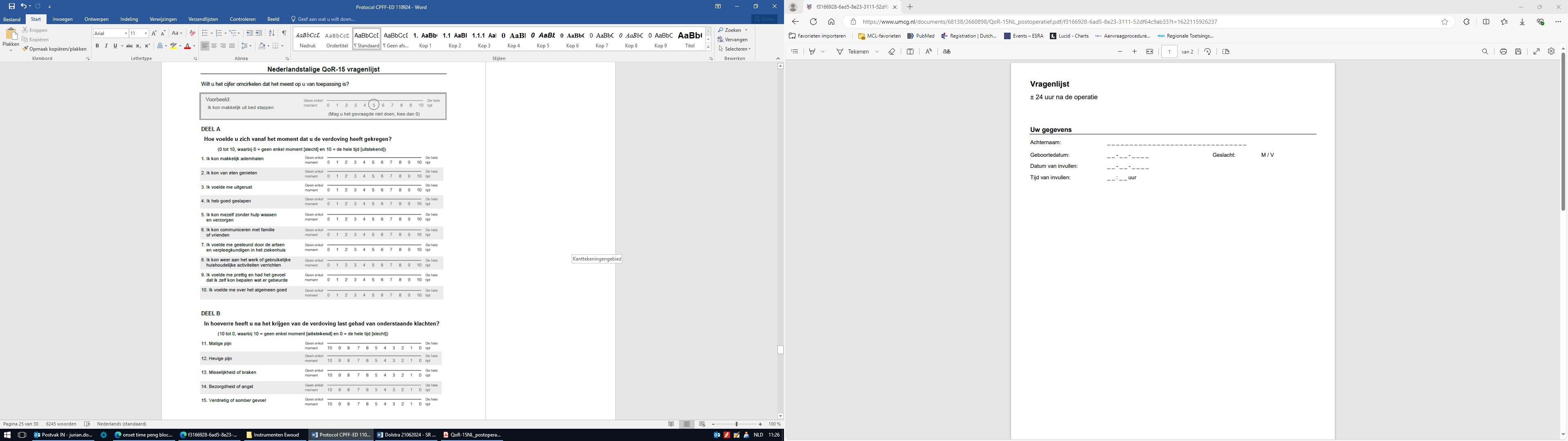
Controleer alstublieft of u **alle** 15 vragen heeft beantwoord. Bedankt voor uw medewerking!

Appendix IV

**Dataverzamelingsformulier***

*Dit formulier zal tijdens de studie niet op papier, maar elektronisch in REDCap zijn.

**Pijnscores rust (VAS)**


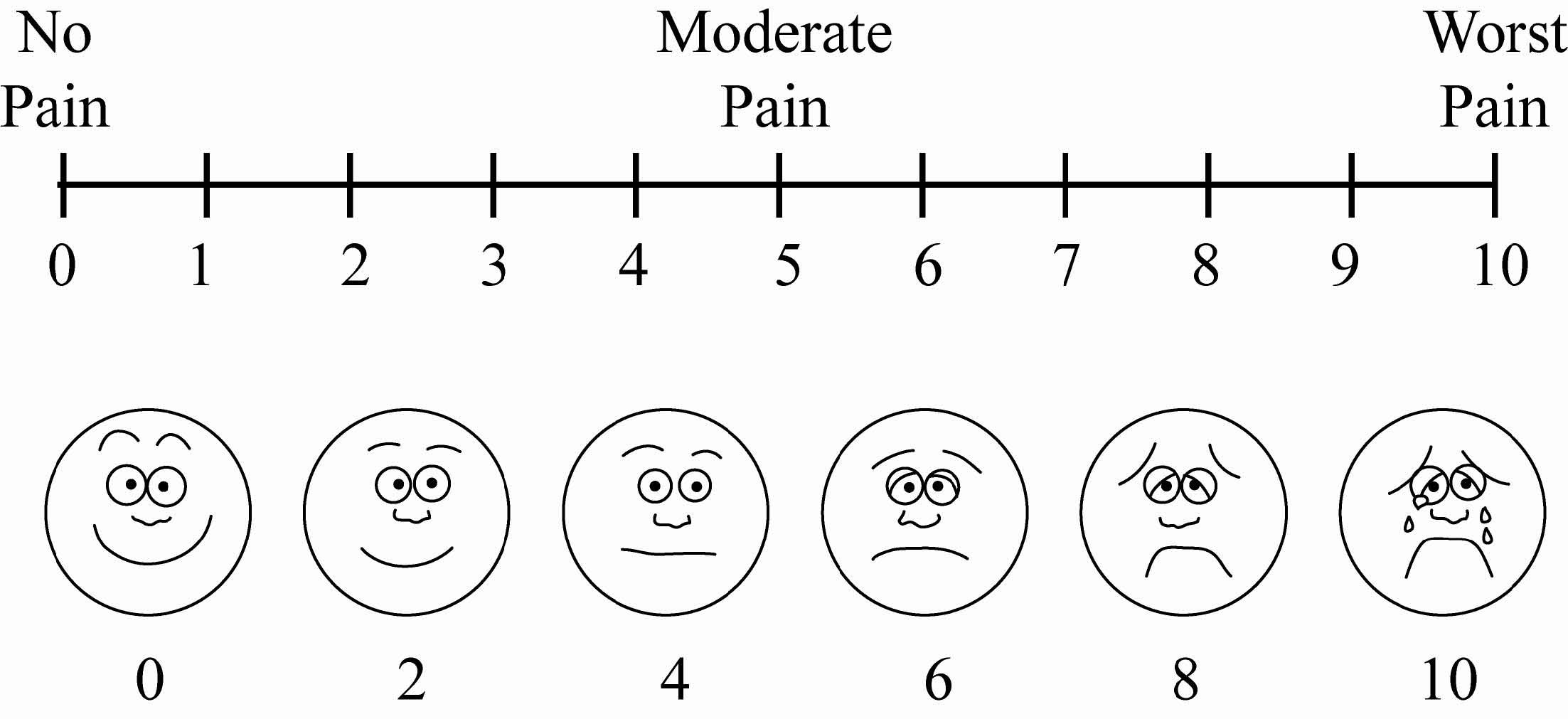
15 min na block :


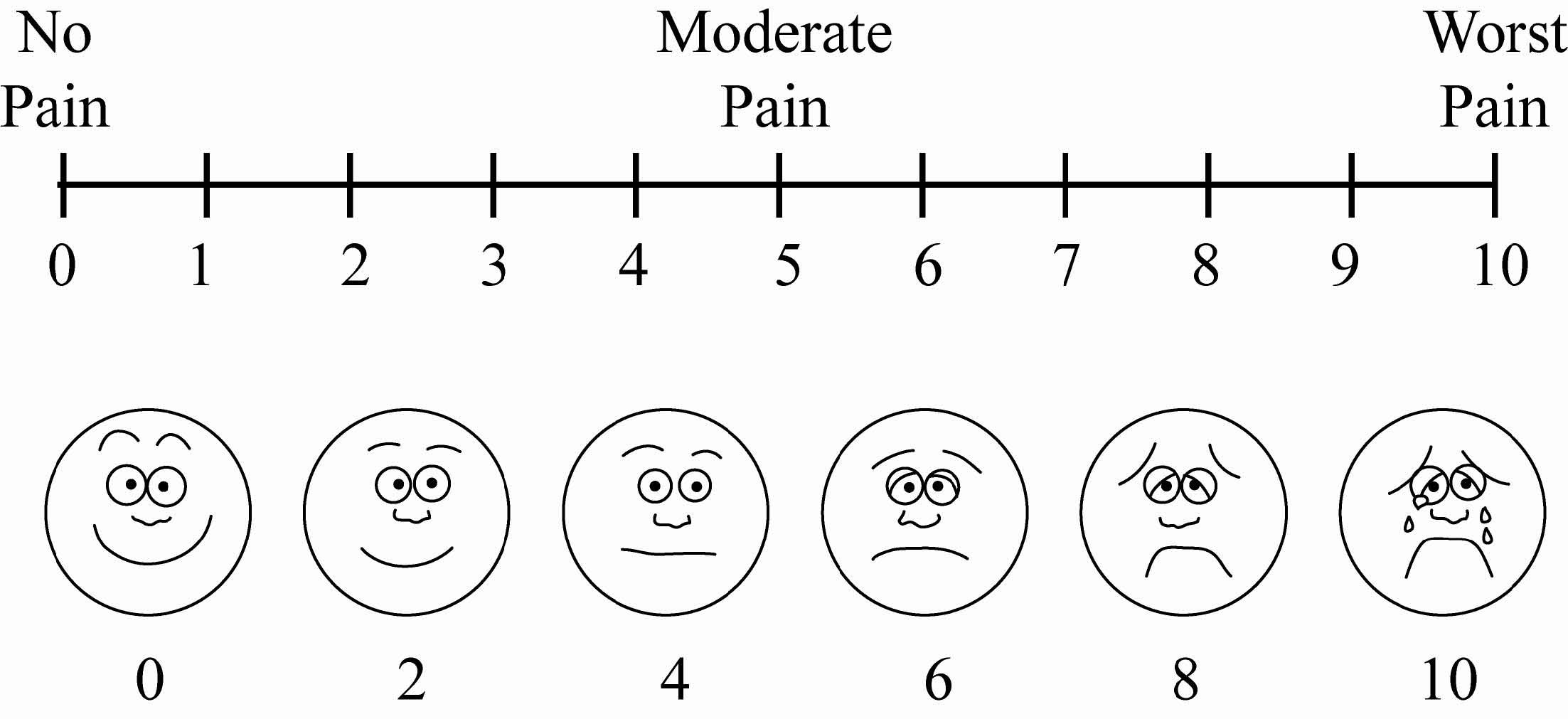
30 min na block :


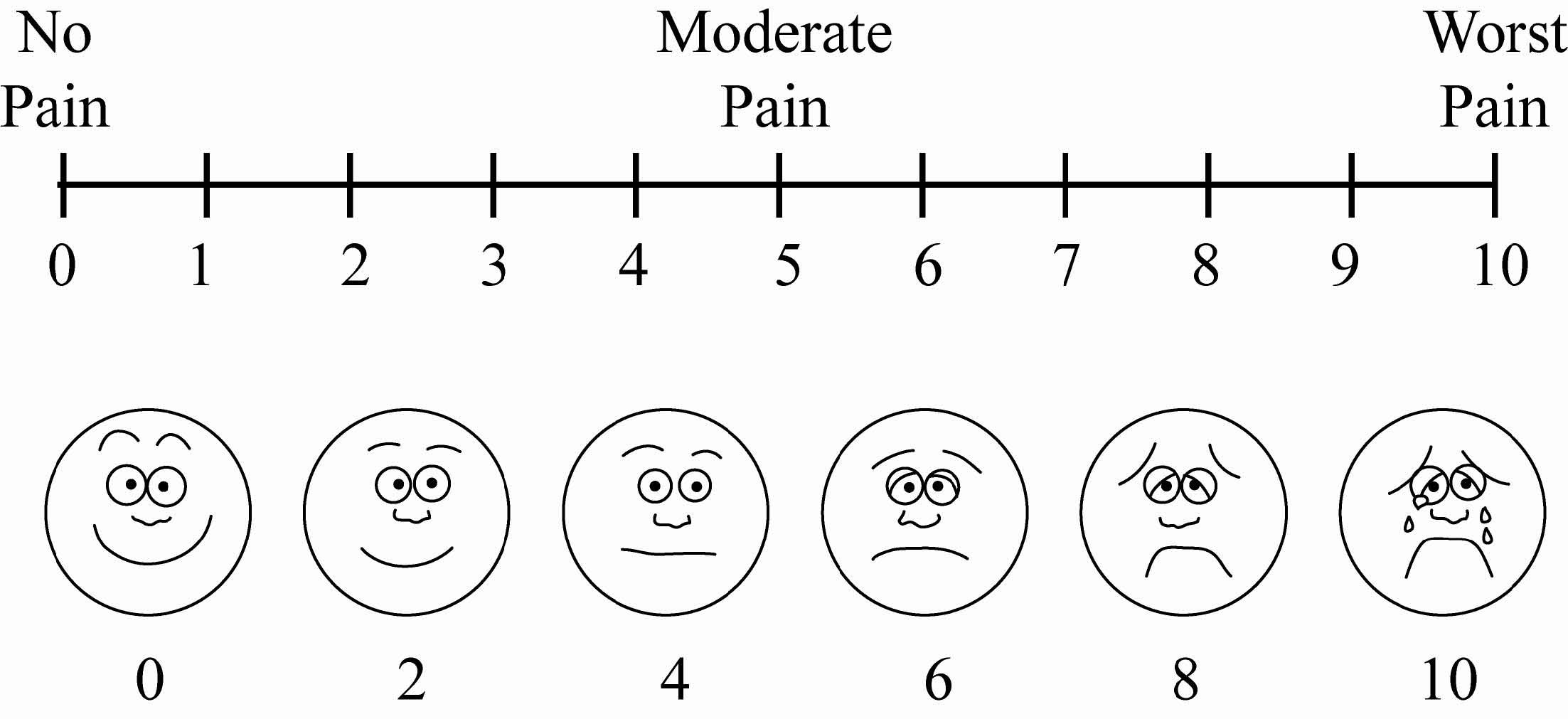
1 uur na block :

**Pijnscores lichte elevatie been (VAS)**


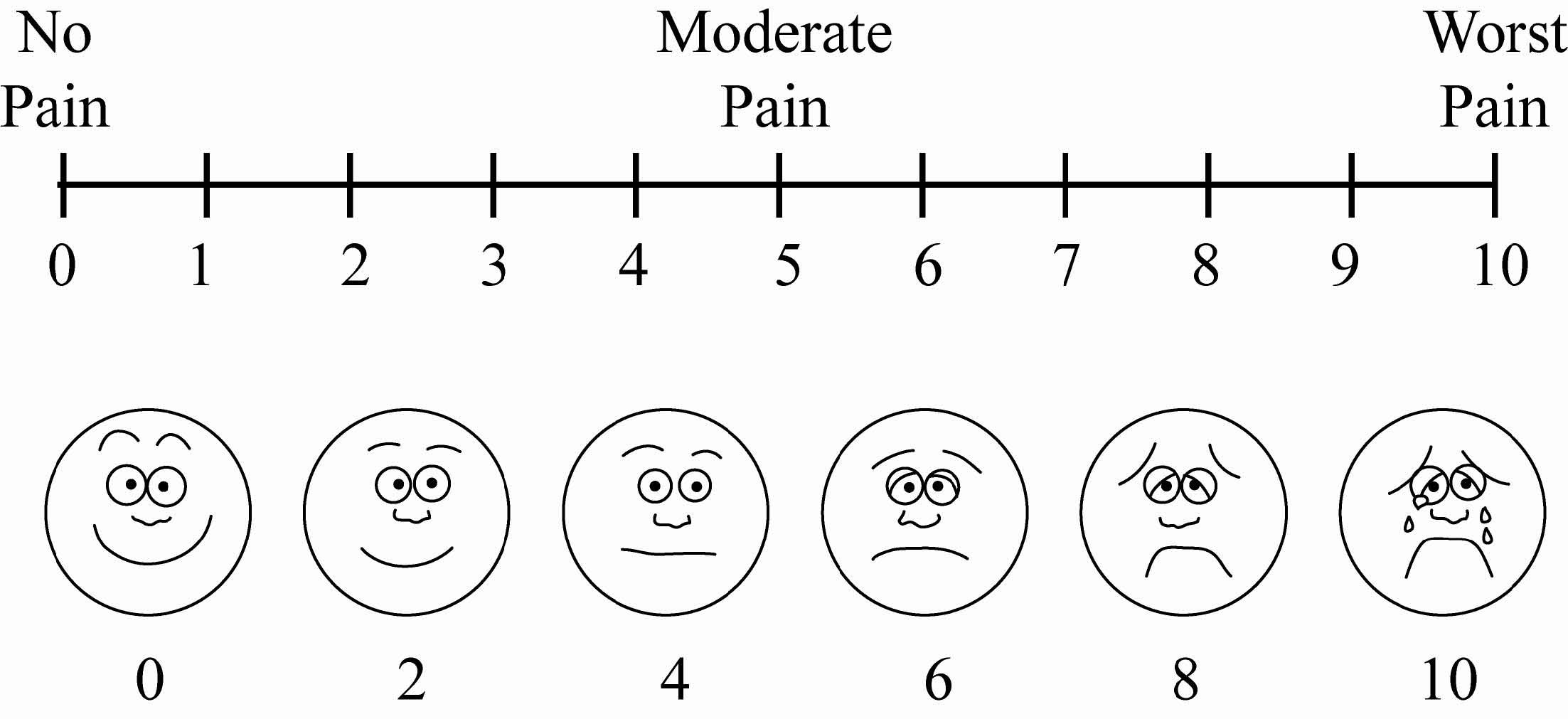
15 min na block :


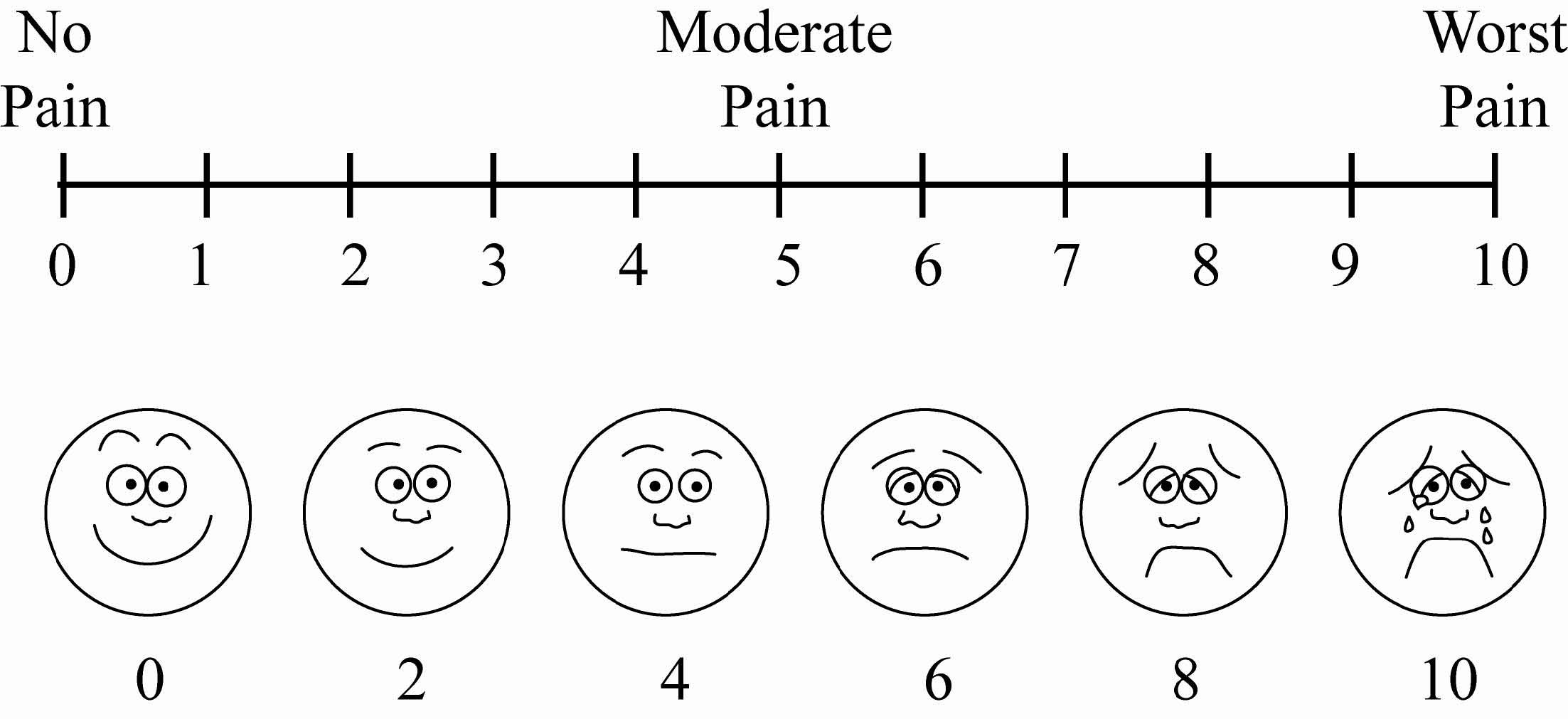
30 min na block :


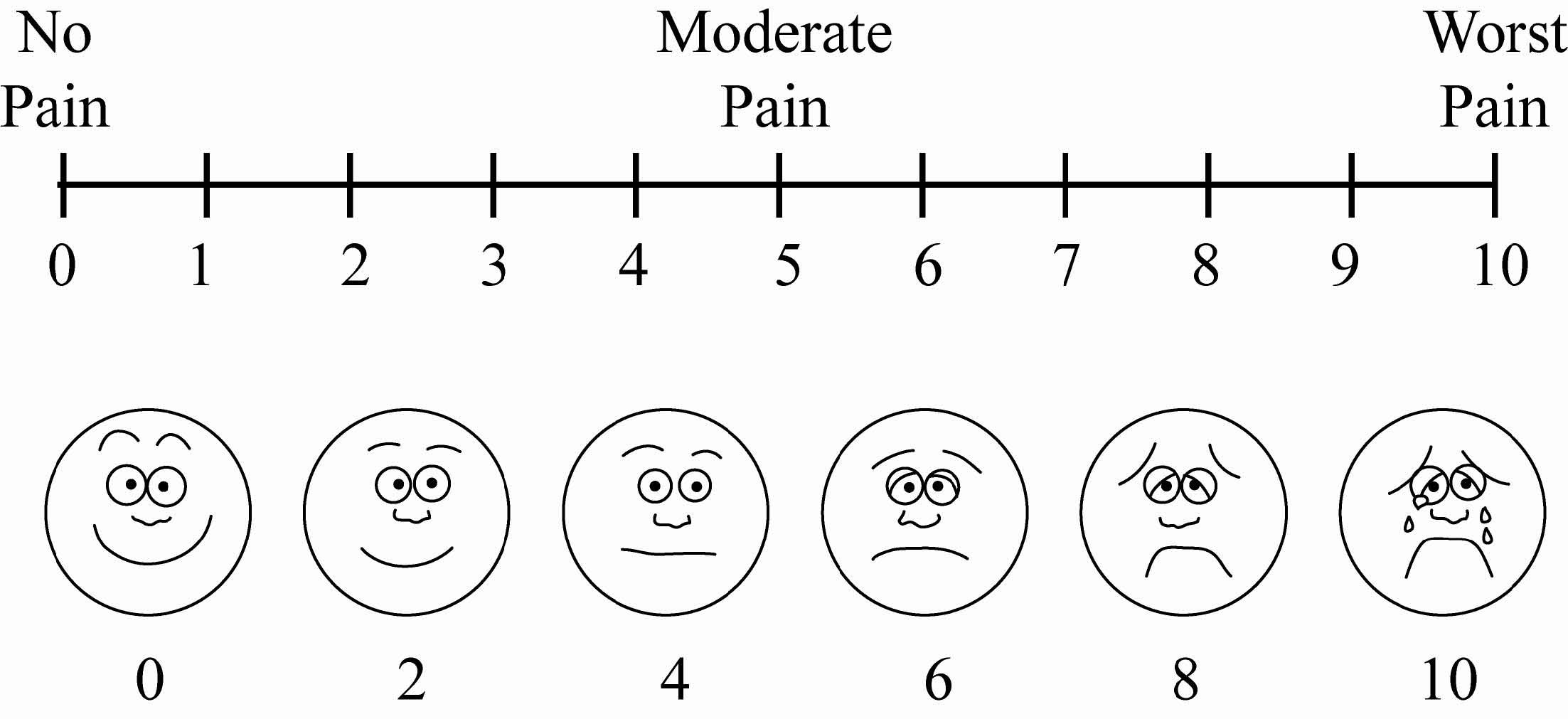
1 uur na block :

**Data te verzamelen vóór block procedure**

Leeftijd : …..…… jaar oud

Geslacht : …..…………………………………………………

Gewicht : …..…… kg

ASA-score : …..…………………………………………………

Type fractuur : …..…………………………………………………

Medicamenten gebruikt vóór ziekenhuis : …..…………………………………………………

..…..…………………………………………………..…..…………………………………………….

..…..…………………………………………………..…..…………………………………………….

Type en dosering anestheticum : …..…………………………………………………

: ……….. ml

Tijdstip plaatsing block : .... : .… uur *(zoals 9:30, op het moment dat het half 10 in de ochtend is)*.

***Zie volgende pagina!***

**Data te verzamelen 12 uur na block procedure**

Opioïden toegediend na block (incl. dosering)

..…..…………………………………………………..…..…………………………………………….

..…..…………………………………………………..…..…………………………………………….

..…..…………………………………………………..…..…………………………………………….

Tijdstip meting opioïden: .... : .… uur *(zoals 9:30, op het moment dat het half 10 in de ochtend is)*.

Hoeveelheid benodigde opioïden: ……. mg/uur

(S)AE’s voorgekomen (omcirkel) : Ja / Nee

Indien “Ja”, specificeer : …..…………………………………………………

..…..…………………………………………………..…..…………………………………………….

..…..…………………………………………………..…..…………………………………………….

Is er sprake van een verband tussen LA en SAE? (omcirkel) Ja / Nee

Appendix V

**Proefpersoneninformatie voor deelname
aan medisch-wetenschappelijk onderzoek**

**CPFF-ED: Een vergelijking van verschillende soorten plaatselijke verdoving voor mensen met een gebroken heup**

*Een vergelijking van pericapsulaire zenuwgroepblokkade, fascia-iliaca compartiment blokkade en femorale zenuwblokkade voor pijnbehandeling bij patiënten met een heupfractuur op de spoedeisende hulp – Een gerandomiseerde gecontroleerde studie.*

**Inleiding**

Geachte heer/mevrouw,

Met deze informatiebrief willen we u vragen of u wilt meedoen aan medisch-wetenschappelijk onderzoek. Meedoen is vrijwillig. U krijgt deze brief omdat u uw heup heeft gebroken.

U leest hier om wat voor onderzoek het gaat, wat het voor u betekent, en wat de voordelen en nadelen zijn. Het is veel informatie. Wilt u de informatie doorlezen en beslissen of u wilt meedoen? Als u wilt meedoen, kunt u het formulier invullen dat u vindt in bijlage C.

Gezien de spoedeisende aard van de (behandeling van de) aandoening zal u 10-20 minuten de tijd krijgen om te besluiten of u mee wilt doen moet dit onderzoek. Deze tijd is korter dan normaal gesproken door de WMO wordt opgelegd. Omdat u vóór de ingreep maar beperkte bedenktijd heeft, wordt u na de ingreep nogmaals gevraagd of u mee wilt doen met dit onderzoek. U kunt uw toestemming dan alsnog intrekken als u dat wilt.

**Stel uw vragen**

U kunt uw beslissing nemen met de informatie die u in deze informatiebrief vindt. Daarnaast raden we u aan om dit te doen:

- Stel vragen aan de onderzoeker die u deze informatie geeft.

- Praat met uw partner, familie of vrienden over dit onderzoek.

- Stel vragen aan de onafhankelijk deskundige. Voor contactgegevens zie bijlage A.

- Lees de informatie op [www.rijksoverheid.nl/mensenonderzoek](http://www.rijksoverheid.nl/mensenonderzoek).

1. **Algemene informatie**

Het Medisch Centrum Leeuwarden (MCL) heeft dit onderzoek opgezet. Hieronder noemen we het MCL steeds de ‘opdrachtgever’. Onderzoekers, dit kunnen ook artsen zijn, voeren het onderzoek uit in verschillende ziekenhuizen.

Deelnemers aan een medisch-wetenschappelijk onderzoek worden vaak proefpersonen genoemd. Zowel patiënten als mensen die gezond zijn, kunnen proefpersoon zijn. Voor dit onderzoek zijn 198 proefpersonen nodig.

De medisch-ethische toetsingscommissie RTPO heeft dit onderzoek goedgekeurd.

**2. Wat is het doel van het onderzoek?**

Het doel van deze studie is om de werkzaamheid en veiligheid van verschillende manieren van plaatselijke verdoving voor mensen met een gebroken heup op de spoedeisende hulp te vergelijken.

1. **Wat is de achtergrond van het onderzoek?**

Onderzoek waarin de verschillende opties voor plaatselijke verdoving na een heupfractuur rechtstreeks worden vergeleken is vooralsnog schaars en niet gericht op de spoedeisende hulp. We willen hier graag meer duidelijkheid in brengen, om patiënten in de toekomst zo goed mogelijk pijnstilling te kunnen bieden.

1. **Hoe verloopt het onderzoek?**

*Hoelang duurt het onderzoek?*

Doet u mee met het onderzoek? We zullen gedurende 24 uur data van u verzamelen. Het afnemen van de vragenlijst zal ongeveer 5-10 minuten in beslag nemen.

*Stap 1: bent u geschikt om mee te doen?*

We willen eerst weten of u geschikt bent om mee te doen. Daarvoor zal de onderzoeker een aantal dingen onderzoeken:

- Bent u 18 jaar of ouder?
- Wat voor breuk heeft u?
- Heeft u een kunstheup?
- Bent u allergisch voor het verdovingsmiddel dat gebruikt zal worden?
- Is er sprake van een infectie of huidaandoening die de injectie of het gebruik van de echoapparatuur hindert?

*Stap 2: de behandeling.*

We behandelen uw pijn met plaatselijke verdoving. Hiervoor zijn 3 opties. Alle 3 deze opties zijn al onderdeel van de gewone zorg.

Voor dit onderzoek maken we dan ook 3 groepen:

- Groep 1. De mensen in deze groep krijgen het Femoraal Blok (FNB).
- Groep 2. De mensen in deze groep krijgen Fascia-Iliaca Compartiment Blok (FICB).
- Groep 3. De mensen in deze groep krijgen het Periscapulair Zenuwgroep Blok (PENG).

Loting bepaalt in welke groep u zal zitten. U en de onderzoeker weten niet in welke groep u zit. Als het voor uw gezondheid belangrijk is, kan dit wel worden opgezocht. De arts weet wel in welke groep u zit. Dit is noodzakelijk voor de behandeling.

*Stap 3: onderzoeken en metingen*

Het onderzoek plaatsvinden gedurende uw opname op de spoedeisende hulp. U hoeft voor het onderzoek dus niet vaker naar het ziekenhuis te komen. We doen de volgende (hoofd)onderzoeken:

- We zullen op verschillende momenten vragen en noteren hoe veel pijn u heeft, aan de hand van een score.
- Na 6 tot 8 uur, of voordat u geopereerd zal worden, zal de onderzoeker bij u langs komen of u bellen om een vragenlijst in te vullen. De vragen gaan over de periode die volgde op de verdoving. Het invullen van de vragenlijst duurt ongeveer 5-10 minuten.
- We zullen bijhouden hoeveel andere medicatie u gebruikt gedurende het onderzoek.
- We zullen bijhouden of er complicaties optreden.

*Wat is er anders dan bij gewone zorg?*

Er is bij dit onderzoek niet zoveel anders dan bij gewone zorg. De verschillende manieren van verdoving en ook het gebruik van het echoapparaat zijn namelijk al onderdeel van de gewone zorg. De controles van uw pijn en de vragenlijst die bij dit onderzoek horen, zijn extra.

1. **Welke afspraken maken we met u?**

We willen graag dat het onderzoek goed verloopt. Daarom maken we de volgende afspraken met u:

- U doet mee aan het onderzoek op de manier die de onderzoeker u heeft uitgelegd.
- U doet tijdens dit onderzoek niet mee aan een ander medisch-wetenschappelijk onderzoek.
- U neemt contact op met de onderzoeker in deze situaties:
  - U wilt andere medicijnen gaan gebruiken. Ook als dit homeopathische middelen, natuurgeneesmiddelen, vitaminen of geneesmiddelen van de drogist zijn.
  - U krijgt plotseling problemen met uw gezondheid.
  - U wilt niet meer meedoen met het onderzoek.

1. **Van welke bijwerkingen, nadelige effecten of ongemakken kunt u last krijgen?**

Het onderzoeksmiddel kan bijwerkingen/nadelige effecten geven. Geen van deze bijwerkingen komen vaak voor.

Let op: tijdens of na het aanbrengen van de verdoving, kan in zeer uitzonderlijke situaties een deel van de verdovingsvloeistof in uw bloed terecht komen. Een teken hiervan is een metaalachtige smaak in de mond. Waarschuw meteen de arts en/of onderzoeker als u zich tijdens of kort na de procedure bewust wordt van het proeven van een metaalsmaak.

De volgende bijwerkingen worden geassocieerd met plaatselijke verdoving:

- - Een vervelend gevoel op de plek van de injectie.
  - Een tintelend gevoel.
  - Oorsuizen.
  - Hoofdpijn, duizeligheid, minder scherp zicht of verwarring.
  - Spierkrampen of trillen.

De volgende bijwerkingen van plaatselijke verdoving komen enorm weinig voor, maar kunnen ernstig zijn:

- - Vergiftiging door de plaatselijke verdoving (Local Anaesthetic Systemic Toxicity; LAST).
  - Bloeding door in het femorale bloedvat te prikken.
  - Zenuwschade.
  - Anafylaxie; overgevoeligheidsreactie.
  - Hartstilstand.
  - Hartritmestoornis.
  - Lage bloeddruk.

De methode die we onderzoeken kan ook bijwerkingen hebben die we nu nog niet weten.

1. **Wat zijn de voordelen en de nadelen als u meedoet aan het onderzoek?**

Meedoen aan het onderzoek kan voordelen en nadelen hebben. Hieronder zetten we ze op een rij. Denk hier goed over na, en praat erover met anderen.

Meedoen aan het onderzoek kan deze voordelen hebben:

- - Door uw deelname zullen patiënten met dezelfde klachten in de toekomst beter geholpen kunnen worden.

Meedoen aan het onderzoek kan deze nadelen of gevolgen hebben:

- - Meedoen aan het onderzoek kost u extra tijd.

*Wilt u niet meedoen?*

U beslist zelf of u meedoet aan het onderzoek. Wilt u niet meedoen? Dan krijgt u de gewone behandeling voor uw gebroken heup. De gewone behandeling is in dit geval middels dezelfde plaatselijke verdoving als in het onderzoek.

Echter zal het type verdoving dat u krijgt dan niet via loting bepaald worden, zullen we dan niet vragen naar uw pijn, en hoeft u geen vragenlijst in te vullen.

1. **Wanneer stopt het onderzoek?**

De onderzoeker laat het u weten als er nieuwe informatie over het onderzoek komt die belangrijk voor u is. De onderzoeker vraagt u daarna of u blijft meedoen.

In deze situaties stopt voor u het onderzoek:

- Alle benodigde gegevens zoals beschreven zijn verzameld.
- U wilt zelf stoppen met het onderzoek. Dat mag op ieder moment. Meld dit dan meteen bij de onderzoeker. U hoeft er niet bij te vertellen waarom u stopt.
- De onderzoeker vindt het beter voor u om te stoppen.
- Een van de volgende instanties besluit dat het onderzoek moet stoppen:
  - Het MCL,
  - de overheid, of
  - de medisch-ethische commissie die het onderzoek beoordeelt.

*Wat gebeurt er als u stopt met het onderzoek?*

De onderzoekers gebruiken de gegevens die tot het moment van stoppen zijn verzameld.

1. **Wat gebeurt er na het onderzoek?**

*Krijgt u de resultaten van het onderzoek?*

Ongeveer een jaar nadat het onderzoek is afgerond laat de onderzoeker u weten wat de belangrijkste uitkomsten zijn van het onderzoek. De onderzoeker kan u ook vertellen in welke groep u zat. Wilt u dit niet weten? Zeg dat dan tegen de onderzoeker. Hij/zij zal het u dan niet vertellen.

1. **Wat doen we met uw gegevens?**

Doet u mee met het onderzoek? Dan geeft u ook toestemming om uw gegevens te verzamelen, gebruiken en bewaren.

*Welke gegevens bewaren we?*

We bewaren deze gegevens:

- uw naam

- uw geslacht

- uw adres

- uw geboortedatum

- uw gewicht

- gegevens over uw gezondheid

- (medische) gegevens die we tijdens het onderzoek verzamelen

*Waarom verzamelen, gebruiken en bewaren we uw gegevens?*

We verzamelen, gebruiken en bewaren uw om de vragen van dit onderzoek te kunnen beantwoorden en om de resultaten te kunnen publiceren.

*Hoe beschermen we uw privacy?*

Om uw privacy te beschermen geven wij uw gegevens een code. Op al uw gegevens zetten we alleen deze code. De sleutel van de code bewaren we op een beveiligde plek in het ziekenhuis. Als we uw gegevens verwerken, gebruiken we steeds alleen die code. Ook in rapporten en publicaties over het onderzoek kan niemand terughalen dat het over u ging.

*Wie kunnen uw gegevens zien?*

Sommige personen kunnen wel uw naam en andere persoonlijke gegevens zonder code inzien. Dit kunnen gegevens zijn die speciaal voor dit onderzoek zijn verzameld, maar ook gegevens uit uw medisch dossier.

Dit zijn mensen die controleren of de onderzoekers het onderzoek goed en betrouwbaar uitvoeren. Deze personen kunnen bij uw gegevens komen:

- Nationale en internationale toezichthoudende autoriteiten.

Deze personen houden uw gegevens geheim. Voor inzage door deze personen vragen wij u toestemming te geven. De Inspectie Gezondheidszorg en Jeugd kan zonder uw toestemming uw gegevens inzien.

*Hoelang bewaren we uw gegevens en lichaamsmateriaal?*

We bewaren uw gegevens 10 jaar in het ziekenhuis.

*Mogen we uw gegevens gebruiken voor ander onderzoek?*

Uw verzamelde gegevens kunnen ook van belang zijn voor ander wetenschappelijk onderzoek op het gebied van plaatselijke verdoving bij heupfracturen. Uw gegevens zullen 10 jaar worden bewaard in het ziekenhuis en in het toestemmingformulier geeft u aan of het goed vindt dat uw data in ander onderzoek gebruikt kan worden. Geeft u geen toestemming? Dan kunt u nog steeds meedoen met dit onderzoek. U krijgt dezelfde zorg.

*Kunt u uw toestemming voor het gebruik van uw gegevens weer intrekken?*

U kunt uw toestemming voor het gebruik van uw gegevens op ieder moment intrekken. Zeg dat dan tegen de onderzoeker. Dit geldt voor het gebruik in dit onderzoek en voor het gebruik in ander onderzoek. Maar let op: trekt u uw toestemming in, en hebben onderzoekers dan al gegevens verzameld voor een onderzoek? Dan mogen zij deze gegevens nog wel gebruiken.

*Wilt u meer weten over uw privacy?*

- Wilt u meer weten over uw rechten bij de verwerking van persoonsgegevens? Kijk dan op [www.autoriteitpersoonsgegevens.nl](http://www.autoriteitpersoonsgegevens.nl).
- Heeft u vragen over uw rechten? Of heeft u een klacht over de verwerking van uw persoonsgegevens? Neem dan contact op met degene die verantwoordelijk is voor de verwerking van uw persoonsgegevens. Voor uw onderzoek is dat:
  - Medisch Centrum Leeuwarden. Zie bijlage A voor contactgegevens, en website.
- Als u klachten heeft over de verwerking van uw persoonsgegevens, raden we u aan om deze eerst te bespreken met het onderzoeksteam. U kunt ook naar de Functionaris Gegevensbescherming van het MCL gaan. Of u dient een klacht in bij de Autoriteit Persoonsgegevens.

*Waar vindt u meer informatie over het onderzoek?*

Op de volgende website(s) vindt u meer informatie over het onderzoek: www.onderzoekmetmensen.nl . Na het onderzoek kan de website een samenvatting van de resultaten van dit onderzoek tonen. U vindt het onderzoek door te zoeken op NL87859.099.24

1. **Krijgt u een vergoeding als u meedoet aan het onderzoek?**

Meedoen aan het onderzoek levert u geen extra kosten op. U krijgt ook geen vergoeding als u meedoet aan dit onderzoek.

1. **Bent u verzekerd tijdens het onderzoek?**

U bent niet extra verzekerd voor dit onderzoek. Want als u meedoet aan het onderzoek, heeft u dezelfde risico’s als bij de gewone behandeling van uw gebroken heup. Daarom hoeft de onderzoeker van de RTPO geen extra verzekering af te sluiten.

1. **Heeft u vragen?**

Vragen over het onderzoek kunt u stellen aan de onderzoeker. Wilt u advies van iemand die er geen belang bij heeft? Ga dan naar de onafhankelijk deskundige, voor contactgegevens zie bijlage A. Hij weet veel over het onderzoek, maar werkt niet mee aan dit onderzoek.

Heeft u een klacht? Bespreek dit dan met de onderzoeker of de arts die u behandelt. Wilt u dit liever niet? Ga dan naar de klachtencommissie van het MCL. In bijlage A staat waar u die kunt vinden.

1. **Hoe geeft u toestemming voor het onderzoek?**

U kunt even nadenken over dit onderzoek. Omdat het om onderzoek op de spoedeisende hulp gaat, en zowel u als wij gebaat zijn bij snelle handeling, krijgt u een aantal minuten om na te denken over uw deelname. Daarna vertelt u de onderzoeker of u de informatie begrijpt en of u wel of niet wilt meedoen. Wilt u meedoen? Dan vult u het toestemmingsformulier in dat u bij deze informatiebrief vindt. U en de onderzoeker krijgen allebei een getekende versie van deze toestemmingsverklaring.

Dank voor uw tijd.

1. **Bijlagen bij deze informatie**

A. Contactgegevens

B. Toestemmingsformulier(en)

**Bijlage A: contactgegevens**

**(Lokale) Hoofdonderzoeker**

Naam: Dr. H. Lameijer

Functie: Spoedeisende Hulp arts MCL

E-mail: heleen.lameijer1@mcl.nl

Telefoonnummer: 058 286 6666

**Uitvoerende onderzoeker:**

Naam: Jurian Dolstra, Msc

Functie: Promovendus Spoedeisende Hulp MCL

E-mail: jurian.dolstra@mcl.nl

Telefoonnummer: 058 286 3684

**Onafhankelijk deskundige:**

Naam: Drs. Tom Boeije

Functie: Spoedeisende Hulp arts Dijklander ziekenhuis

E-mail: jurian.dolstra@mcl.nl

Telefoonnummer: 022 920 8071

**Klachten**

Indien u klachten heeft over het onderzoek, kunt u dit bespreken met de onderzoeker of uw behandelend arts. Wilt u dit liever niet, dan kunt u zich wenden tot de ombudsfunctionaris van het MCL.

Ombudsfunctionaris MCL

Kantoor: Route 78 van het MCL

Telefoonnummer: 058 286 7031

Bereikbaarheid: kantoortijden

Website: <https://www.mcl.nl/praktische-informatie/rechten-plichten-en-klachten/klachten/ombudsfunctionaris>

Voor algemene informatie over uw rechten bij verwerking van uw persoonsgegevens kunt u de website van de Autoriteit Persoonsgegevens raadplegen. Bij vragen of klachten over het gebruik of de verwerking van uw gegevens, of over uw rechten, kunt u contact opnemen met:

Naam: Mevrouw E. Klop

Functie: Functionaris Gegevensbescherming Medisch Centrum Leeuwarden

Telefoonnummer: 0582866310

E-mail: [els.klop@mcl.nl](mailto:els.klop@mcl.nl)

**Bijlage B: toestemmingsformulier proefpersoon**

Behorende bij

**CPFF-ED: Een vergelijking van verschillende soorten plaatselijke verdoving voor mensen met een gebroken heup**

- Ik heb de informatiebrief gelezen. Ook kon ik vragen stellen. Mijn vragen zijn goed genoeg beantwoord. Ik had genoeg tijd om te beslissen of ik meedoe.
- Ik weet dat meedoen vrijwillig is. Ook weet ik dat ik op ieder moment kan beslissen om toch niet mee te doen met het onderzoek. Of om ermee te stoppen. Ik hoef dan niet te zeggen waarom ik wil stoppen.
- Ik geef de onderzoekers toestemming om mijn gegevens te verzamelen en gebruiken. De onderzoekers doen dit alleen om de onderzoeksvraag van dit onderzoek te beantwoorden.
- Ik weet dat voor de controle van het onderzoek sommige mensen al mijn gegevens kunnen inzien. Die mensen staan in deze informatiebrief. Ik geef deze mensen toestemming om mijn gegevens in te zien voor deze controle.
- Wilt u in de tabel hieronder ja of nee aankruisen?

| Ik geef toestemming om mijn gegevens te bewaren om dit te gebruiken voor ander onderzoek, zoals in de informatiebrief staat. | Ja ☐ | Nee☐ |
| --- | --- | --- |
| Ik geef de onderzoekers toestemming om na het onderzoek te laten weten welke behandeling ik heb gehad/ in welke groep ik zat. | Ja ☐ | Nee☐ |

- Ik wil meedoen aan dit onderzoek.

Mijn naam is (proefpersoon): ………………………………..

Handtekening: ……………………… Datum : __ / __ / __

-----------------------------------------------------------------------------------------------------------------

Ik verklaar dat ik deze proefpersoon volledig heb geïnformeerd over het genoemde onderzoek.

Wordt er tijdens het onderzoek informatie bekend die die de toestemming van de proefpersoon kan beïnvloeden? Dan laat ik dit op tijd weten aan deze proefpersoon.

Naam onderzoeker (of diens vertegenwoordiger):……………………………….

Handtekening:……………………… Datum: __ / __ / __

-----------------------------------------------------------------------------------------------------------------

*De proefpersoon krijgt een volledige informatiebrief mee, samen met een getekende versie van het toestemmingsformulier.*
